# Mindfulness as a Protective Factor Against Depression, Anxiety and Psychological Distress During the COVID-19 Pandemic: Emotion Regulation and Insomnia Symptoms as Mediators

**DOI:** 10.3389/fpsyg.2022.820959

**Published:** 2022-04-01

**Authors:** André Mamede, Inge Merkelbach, Gera Noordzij, Semiha Denktas

**Affiliations:** ^1^Department of Psychology, Education and Child Development, Erasmus School of Social and Behavioural Sciences, Erasmus University Rotterdam, Rotterdam, Netherlands; ^2^Erasmus University College, Erasmus School of Social and Behavioural Sciences, Erasmus University Rotterdam, Rotterdam, Netherlands

**Keywords:** mindfulness, depression, anxiety, emotion regulation, rumination, sleep, insomnia

## Abstract

**Objectives:**

Research has linked mindfulness to improved mental health, yet the mechanisms underlying this relationship are not well understood. This study explored the mediating role of emotion regulation strategies and sleep in the relationship between mindfulness and symptoms of depression, anxiety and psychological distress during the COVID-19 pandemic.

**Methods:**

As detailed in this study’s pre-registration (osf.io/k9qtw), a cross-sectional research design was used to investigate the impact of mindfulness on mental health and the mediating role of emotion regulation strategies (i.e., cognitive reappraisal, rumination and suppression) and insomnia. A total of 493 participants from the general population answered an online survey and were included in the final analysis. The online survey consisted of the short form of the Five-Facets Mindfulness Questionnaire (FFMQ-SF), the Impact of Event Scale-revised (IES-R), the Generalised Anxiety Disorder Scale (GAD-7), the Patient Health Questionnaire (PHQ-8), the Emotion Regulation Questionnaire (ERQ), the short form of the Rumination Response Scale (RSS-SF), and the Insomnia Severity Index (ISI).

**Results:**

Structural equation modelling revealed that mindfulness was related to lower symptoms of depression, anxiety and psychological distress, both directly and indirectly. Mindfulness was negatively associated with rumination and insomnia. As hypothesised, models revealed that the associations between mindfulness and depression, anxiety and psychological distress were significantly mediated by its negative associations with rumination and insomnia. Our findings also demonstrated that rumination was related to increased insomnia symptoms, which in turn was associated with increased mental health problems, indicating a mediated mediation. Mindfulness was also positively associated with cognitive reappraisal and negatively associated with suppression, which were, respectively, negatively and positively associated with depressive symptoms, and thus functioned as specific mediators of the association between mindfulness and depression.

**Conclusion:**

Our findings suggest that rumination and insomnia operate transdiagnostically as interrelated mediators of the effects of mindfulness on mental health, whereas cognitive reappraisal and suppression function as specific mediators for depression. These insights emphasise the importance of targeting emotion regulation and sleep in mindfulness interventions for improving mental health. Limitations and implications for practice are discussed.

## Introduction

The global outbreak of the coronavirus disease 2019 (COVID-19) was declared a pandemic by the World Health Organization (WHO) on March 11th of 2020 (Mahase, 2020), leading to the implementation of social isolation measures throughout the world. Besides social isolation, most people have had to deal with other serious challenges, such as financial worries and health concerns. Consequently, research has reported alarming rates of anxiety (20–59%), depression (25–46%), and stress (34–70%) in the general population during the COVID-19 pandemic (Burke et al., 2020; Rodríguez-Rey et al., 2020; Vindegaard and Benros, 2020; Wang et al., 2020a; Varma et al., 2021). Such rates are considerably higher than 12 month prevalences for anxiety (10.6%) and depression (9.6%) reported in pre-pandemic epidemiological studies (Mojtabai et al., 2016; Remes et al., 2016). On the other hand, some studies demonstrated that certain individuals were less vulnerable to experiencing mental health problems during the pandemic (Killgore et al., 2020; Cunningham et al., 2021; Fields et al., 2021). It is well established that certain people are more vulnerable to mental health problems in highly stressful situations, whereas others seem incredibly resilient in the face of adversity. Therefore, besides monitoring individuals’ mental health during the crisis, it is also crucial to identify modifiable risk and protective factors that can be targeted through cost-effective interventions to prevent mental health problems, both in times of adversity and in daily life. One of such factors is mindfulness, a state of nonjudgemental awareness of one’s internal and external experiences in the present moment (Baer, 2003). Mindfulness has been identified as a buffer against the negative psychological effects of increased stress and as a general protective factor against mental health problems, which may thus have a particularly robust effect in times of adversity. However, in order to develop more effective mindfulness-based interventions (MBIs) and to understand who would benefit the most from them, it is also critical to investigate the cognitive and behavioural mechanisms underlying the associations between mindfulness and mental health. Additionally, the core constructs of mindfulness and how to measure them are not yet sufficiently understood.

### Mindfulness as a Protective Factor Against Mental Health Problems

Over the last two decades, mindfulness has received increased attention for its positive influence on mental health and as a buffer against the negative psychological effects of highly stressful situations. Mindfulness meditation is the practice of cultivating states of mindfulness, wherein practitioners are taught to focus attention on the breath, bodily sensations and eventually on any object (i.e., thoughts, feelings, but also sounds and other sensory experiences) that appear in conscious awareness. Mindfulness has also been conceptualised as a disposition or trait, indicating one’s tendency to evoke the state of mindfulness in daily life, outside of mindfulness meditation practice (Baer et al., 2006). Besides having been correlated with wellbeing, better interpersonal relations, less burnout and greater job and life satisfaction (Mesmer-magnus et al., 2017), trait mindfulness has been inversely associated with symptoms of depression, anxiety and stress (Soysa and Wilcomb, 2015).

Evidence has shown that MBIs can increase one’s predisposition to be mindful in everyday life (i.e., mindfulness trait) by regularly cultivating state mindfulness through meditation (Gu et al., 2015), which mediated improvements in psychological symptoms. This finding opens new, potentially more promising, avenues for prevention and treatment of anxiety and mood disorders, as well as for the promotion of mental health in the general population. Recently, meta-analysis of MBIs has demonstrated that they were moderately effective at treating anxiety (Hedges’s *g* = 0.63) and depressive symptoms (Hedges’s *g* = 0.59; Hofmann et al., 2010), while another meta-analysis has found that the effects of mindfulness-based interventions for various psychiatric disorders were equivalent or superior to other evidence-based treatments (Goldberg et al., 2021).

Despite the bulk of evidence supporting the efficacy of MBIs and the increased interest in its therapeutic potentials, it is not yet well understood what elements of MBIs and what aspects of mindfulness are responsible for improvements in psychological health. To further improve the efficacy and delivery of MBIs, it is important to address the question of how these interventions are bringing about change. To that end, it is important to not only investigate which mechanisms underlie the link between trait mindfulness and mental health problems, but also to examine which aspects of mindfulness are most strongly related to mental health and these key mechanisms. Such examination allows us to improve our understanding of which target constructs of mindfulness were critical in changing treatment outcomes in MBIs and to refine future interventions and measurement instruments accordingly.

Several different theories of mindfulness and corresponding measurement instruments have been outlined in the literature (for review see Sauer et al., 2013). Based on existing self-report measures of mindfulness, Baer et al. (2006) conducted a factor analysis which yielded five distinct but correlated facets of mindfulness: (1) *Observing*, defined as attending to internal and external experience; (2) *Describing*, defined in terms of labelling internal experience with language; (3) *Acting with awareness*, defined as attending to one’s activities in the moment and as the opposite of acting on automatic pilot; (4) *Nonjudgement of inner experience*, defined in terms of adopting a nonevaluative stance towards thoughts and feelings; and (5) *Nonreactivity to inner experience*, defined as the tendency to allow thoughts and feelings to come and go, without getting caught up or carried away by them (Baer et al., 2008). Based on these findings, the five-facet mindfulness questionnaire (FFMQ) was created. Research has found that the facets *acting with awareness* and *nonjudgement* were stronger predictors of decreased anxiety and depression than other facets of mindfulness (Bohlmeijer et al., 2011; Veehof et al., 2011). Given that different facets of mindfulness may have unique influences on different psychopathologies, the use of broader multi-factorial measures of mindfulness may be essential to better understand how mindfulness facets can differentially protect against depression, anxiety and distress. Besides examining the facets of mindfulness and how to measure them, it is essential to investigate the key mechanisms underlying the link between mindfulness and mental health so that MBIs can be tailored accordingly to potentially increase their effectiveness.

### Mindfulness and Emotion Regulation: Cognitive Reappraisal, Rumination, and Suppression

Recent research has suggested that one potential explanation for the protective effects of mindfulness on mental health is the implementation of more adaptive emotion regulation strategies instead of maladaptive ones. There is ample evidence demonstrating that emotion regulation problems are related with various psychopathologies, but particularly with higher risk of depression and anxiety (Aldao et al., 2010). For example, research has suggested that at least part of the positive effects of mindfulness may be explained by the greater use of adaptive cognitive reappraisal strategies, which has been linked to lower risk of anxiety and depression (Martin and Dahlen, 2005). Cognitive reappraisal involves reinterpreting the meaning of certain situations or stimuli so as to modify one’s initial emotional responses (typically negative) to the experience. The tendency of mindful individuals to evoke nonevaluative awareness of experiences may not only be a form of reappraisal in itself, but such nonjudgemental awareness could also further facilitate the recognition and reinterpretation of negative thoughts and feelings (Desrosiers et al., 2013).

Mindfulness may also influence mental health by reducing the use of maladaptive emotion regulation strategies, such as rumination and suppression. Rumination is characterised by repetitive negative self-critical questioning of one’s thoughts, emotions or circumstances, and, although it can occur as a normal part of human experience, it can be particularly dysfunctional when it is excessive and unmanageable (Treynor et al., 2003). Remarkably, trait mindfulness seems be negatively associated with rumination, possibly because the states of non-judgmental awareness and acceptance associated with mindfulness facilitate the recognition and regulation of negative cognitive patterns characteristic of rumination (Raes and Williams, 2010). Similarly, given the link between mindfulness and acceptance, mindfulness also has been thought to be negatively associated with expressive suppression, an emotion regulation strategy that involves the inhibition of behaviours associated with emotional responses (e.g., facial expressions) and is widely regarded as maladaptive. The use of both expressive suppression and rumination have been consistently linked to higher rates of anxiety and depression (Aldao et al., 2010).

Given that certain emotion regulation strategies may contribute uniquely or transdiagnostically to different psychopathologies, these emotion regulation strategies may partially explain the distinct and common mechanisms through which mindfulness influences risk of depression, anxiety and psychological distress. In support of this notion, a recent cross-sectional study found that the relationship between mindfulness and depression was mediated by its positive association with cognitive reappraisal and by its negative association with suppression and rumination. However, only reappraisal and rumination, but not suppression, mediated the effects of mindfulness on anxiety (Parmentier et al., 2019). Yet, another cross-sectional study found that rumination and reappraisal mediated the effects of mindfulness on depression, whereas its effect on anxiety was mediated by rumination and worry, but not by reappraisal (Desrosiers et al., 2013). Considering that the evidence comparing these mechanisms is still scarce, it is not clear whether rumination, suppression and reappraisal all mediate the effects of mindfulness on anxiety, depression and stress, or whether certain emotion regulation strategies act as distinct mediators for different psychopathologies.

Furthermore, emotion regulation strategies may also influence mental health indirectly through their effect on health behaviours. Difficulties in emotion regulation, characterised partially by maladaptive rumination and reduced use of adaptive strategies (e.g., cognitive reappraisal), may predisposes individuals to several behavioural problems, such as unhealthy eating, substance abuse and sleep problems (Aldao and Christensen, 2015; Pillai and Drake, 2015). Because emotions reflect tendencies of action and are closely related to physiological, motivational and decision-making processes (Desteno et al., 2013), the strategies used to regulate one’s emotional responses can influence the probability of enacting certain behaviours (for review see Aldao and Christensen, 2015). Despite the impact of emotion regulation on health behaviour, few studies have investigated whether the use of emotion regulation strategies (e.g., rumination) mediates the relationship between mindfulness and health behaviours, which may, in turn, partially explain the link between mindfulness and mental health.

### Mindfulness and Sleep

Mindfulness has been positively associated with physical activity, healthy eating and negatively associated with sleep problems (e.g., insomnia) and alcohol use (Sala et al., 2019), all of which have been linked to mental health (Jané-Llopis and Matytsina, 2006; Lopresti et al., 2013; Khalid et al., 2017). Sleep, in particular, has been shown to be one of the strongest behavioural predictors of mental health (Wickham et al., 2020; Gilchrist et al., 2021), and ample evidence has demonstrated a mutually causal relationship between sleep and anxiety and depressive disorders (Alvaro et al., 2011). Given that sleep is highly susceptible to worry, stress and rumination (Pillai and Drake, 2015), mindfulness may be particularly beneficial for decreasing insomnia symptoms and improving sleep behaviour, since the nonjudgemental awareness characteristic of mindfulness has been shown to reduce stress and lessen engagement with worries and ruminative thoughts (Evans and Segerstrom, 2011; Petrocchi and Ottaviani, 2016; Lu et al., 2019). In line with this notion, previous studies have demonstrated that MBIs led to improvements in sleep quality, which were mediated by reductions in rumination (Greeson et al., 2018) and stress (Carlson and Garland, 2005). Additionally, a meta-analysis has found that MBIs were effective in reducing insomnia symptoms (Wang et al., 2020b). These findings indicate that mindfulness can reduce maladaptive emotion regulation (i.e., rumination) and buffer against stress, thereby reducing insomnia symptoms.

Given the well-established link between sleep and mental health, this interplay between improved emotion regulation and sleep may be an important mechanism through which mindfulness can prevent and treat mental health problems. Remarkably, researchers recently found that mindfulness buffered the impact of COVID-19-related stressors on sleep duration (Zheng et al., 2020). Considering that the disruption of routine and high levels of stress in times of adversity may be particularly detrimental to sleep behaviour (Hall et al., 2000; Arora and Grey, 2020) and has shown to increase insomnia symptom during the COVID-19 pandemic (Abdulah and Musa, 2020), sleep may be the most important behavioural mechanism underlying the effect of mindfulness on mental health during such times of crisis. However, no studies thus far have investigated whether this interplay between sleep and rumination mediates the relationship between mindfulness and mental health, which may be particularly relevant during highly stressful times such as the COVID-19 pandemic.

### The Present Study

The COVID-19 pandemic and its repercussions have caused substantial increases in levels of depression, anxiety and psychological distress (Wang et al., 2019, 2020a; Rodríguez-Rey et al., 2020; Vindegaard and Benros, 2020; Varma et al., 2021). In Netherlands, two studies have observed increases in mental health symptoms in the general population during the COVID-19 lockdown that started on the 15th of March of 2020 (Pan et al., 2021; Vloo et al., 2021), whereas another study observed little change in rates of mental health problems of students (van Zyl et al., 2021). Certain protective factors, such as mindfulness and its facets, may reduce vulnerability to psychopathologies and partially explain the heterogeneity of findings regarding mental health outcomes during the pandemic. In face of this crisis, it is important to improve our understanding of the core facets of mindfulness with regards to mental health and the mechanisms underlying the relationship between mindfulness and mental health. To that end, this study firstly examined how mindfulness trait and its facets influence three mental health outcomes, namely, symptoms of depression, anxiety and COVID-19-related psychological distress. Secondly, this study examined whether emotion regulation strategies (i.e., cognitive reappraisal, rumination and suppression) and insomnia mediate the relationship between mindfulness and mental health during the COVID-19 pandemic, as well as whether these mediators operate transdiagnostically or specifically for depression, anxiety and COVID-19-related psychological distress. Finally, we investigated whether the relationship between mindfulness and insomnia was mediated by rumination. We hypothesised that mindfulness trait will be negatively associated with symptoms of mental health problems. We expect that more mindful individuals will use more adaptive (i.e., cognitive regulation) and less maladaptive (i.e., rumination and suppression) emotion regulation strategies, which in turn will be associated with less mental health problems and will mediate the relationship between mindfulness and the studied outcomes. Considering the vital role of sleep in mental health, we expected that mindfulness will be negatively associated with insomnia, which will mediate the negative relationship between mindfulness and the three studied mental health outcomes. Finally, in light of the evidence linking rumination to poor sleep, we hypothesised that rumination would mediate the negative relationship between mindfulness and insomnia.

## Materials and Methods

### Participants and Procedure

From the 30th of May until the 20th of July of 2020, potential participants were invited to participate in a survey-study about the impact of the COVID-19 epidemic on mental health. Students from the Erasmus University of Rotterdam were initially recruited through online advertisements to participate in this study in exchange for study credits. Additionally, through social media posts, potential participants from the general population were also invited to participate, although no compensation was provided. At the end of the survey, participants were asked to share the survey with their social network and invite others who were interested in participating (i.e., snow-ball sampling). Participants were eligible to participate if they were at least 16 years old, spoke fluent English and provided informed consent for their participation. This study was approved by the Ethics Review Committee of the Department of Psychology, Education and Child Studies, Erasmus University Rotterdam (application number 20-051).

### Sample Size

We determined that a minimum sample size of 526 participants was needed for investigating the pre-specified model structure, based on an *a-priori* power calculation based on a small-medium effect size of 0.20, 0.90 estimated power and 0.05 probability level, performed through https://www.danielsoper.com/statcalc/calculator.aspx?id=89. As described in this study’s pre-registration, considering the sensitive time frame of the study and that we expected to exclude 5–10% of survey respondents, we intended to stop data collection once we obtained 580 complete responses to the questionnaire, or by July 20th. Data collection was stopped at July 20th, and after exclusion of incomplete and invalid responses, data were collected from 493 participants.

### Measures

#### Independent Variable

##### Five-Facets Mindfulness Questionnaire-Short Form

Mindfulness was assessed with the short form of the Five-Facets Mindfulness Questionnaire (FFMQ-SF; Bohlmeijer et al., 2011). The FFMQ-SF is a 24-item validated questionnaire that asks to rate the degree to which each statement is true for them, and items are rated on a 5-point Likert scale (from 1 for ‘never or very rarely true’ to 5 for ‘very often or always true’). The FFMQ-SF has been found to have high internal reliability and measures five factors of mindfulness, namely, *observing* (alpha = 0.81), *describing* (alpha = 0.87), *acting with awareness* (alpha = 0.83), *nonjudging* (alpha = 0.83), and *nonreactivity* (alpha = 0.75; Bohlmeijer et al., 2011). Due to the conflicting findings in the literature regarding the factor structure of the FFMQ, particularly regarding the role of the *observing* facet (Bohlmeijer et al., 2011), we will investigate the factor structure of the FFMQ-SF before proceeding to the main analysis.

#### Outcome Measures

##### Impact of Event Scale-Revised

The psychological distress related to the COVID-19 pandemic was assessed with the Impact of Event Scale-revised (IES-R; Weiss and Marmar, 1997). The IES-R consists of 22 statements about feelings towards a specific event (i.e., COVID-19 pandemic), which are rated on a 5-point Likert scale indicating the extent to which participants relate to them (from 0 for ‘not at all’ to 4 for ‘extremely’). The IES-R has a high test–retest reliability ranging from 0.89 to 0.94 and its validity has been demonstrated (Weiss and Marmar, 1997).

##### Patient Health Questionnaire

The Patient Health Questionnaire-8 (PHQ-8; Kroenke et al., 2009) was administered to measure clinical symptoms of depression. The PHQ-8 is an eight-item self-report scale in which participants are asked to rate how often; in the past 2 weeks, they have been bothered by several symptoms of depression. Items are rated on a 4-point scale, ranging from 0 for ‘not at all’ to 3 for ‘nearly every day’. The PHQ-8 has good reliability (alpha = 0.88) and has been validated as a screening tool for depressive symptoms (Kroenke et al., 2009; Shin et al., 2019).

##### Generalised Anxiety Disorder Scale

The Generalised Anxiety Disorder Scale (GAD-7; Löwe et al., 2008) was used to assess symptoms of anxiety. The GAD-7 is a seven-item self-report scale in which participants are asked to rate how often, in the past 2 weeks, they have been bothered by the seven core symptoms of anxiety. Items are rated on a 4-point scale, ranging from 0 for ‘not at all’ to 3 for ‘nearly every day’. The GAD-7 has been shown to have high internal consistency (alpha = 0.89) and its validity for screening of anxiety symptoms in the general population has been demonstrated (Löwe et al., 2008).

#### Mediators

##### Emotion Regulation Questionnaire: Cognitive Reappraisal and Suppression

To assess for the use of certain emotion regulation strategies, namely, cognitive reappraisal and suppression, the Emotion Regulation Questionnaire (ERQ; Gross and John, 2003) was administered. ERQ is a validated and widely used 10-item scale that is divided into two subscales: cognitive reappraisal (six items) and suppression (four items). Cognitive reappraisal subscale includes items such as ‘I control my emotions by changing the way I think about the situation I’m in.’ and the suppression subscale includes items such as, ‘When I am feeling negative emotions, I make sure not to express them’. The ERQ items are rated on a 7-point Likert scale from 1 for ‘strongly disagree’ to 7 for ‘strongly agree’, with higher scores indicating increased use of that strategy.

##### Rumination Response Scale: Rumination

Rumination was assessed by administering the short form of the Rumination Response Scale (RRS-SF; Treynor et al., 2003). The RRS-SF consists of 10 items assessing ruminative tendencies, rated on a 4-point Likert scale indicating how frequent they experienced these ruminative tendencies (from 1 for ‘almost never’ to 4 for ‘almost always’). The RRS-SF has been shown to have a high internal reliability (alpha = 0.85) and its validity has been demonstrated (Erdur-Baker and Bugay, 2010).

##### Insomnia Severity Index

Sleep problems and insomnia were evaluated with the Insomnia Severity Index (ISI; Bastien et al., 2001), which has been validated and shown to be a reliable self-report measure of perceived sleep difficulties (Bastien et al., 2001). The ISI is a brief seven-item screening measure that assesses as: (1) the severity of sleep-onset, (2) sleep maintenance, (3) early morning awakening problems, (4) satisfaction with current sleep pattern, (5) interference with daily functioning, (6) noticeability of impairment attributed to the sleep problem and (7) level of distress caused by the sleep problem (Bastien et al., 2001). Each statement refers to the participant’s experiences during the previous 2 weeks and is rated on a 5-point Likert scale (from 0 for ‘not at all/none’ to ‘4 for very much/severe’). The ISI has excellent internal consistency in both community (alpha = 0.90) and clinical samples (alpha = 0.91; Morin et al., 2011).

#### Covariates

##### Descriptive Statistics

Descriptive Statistics on Sociodemographic Characteristics Were Collected. Participants Were Asked about their Age, Gender, Relationship Status, Nationality, Country of Residency and their Highest Level of Education.

##### COVID-19-Related Experiences

We assessed potentially relevant COVID-19-related experiences including health status (presence of COVID-19 symptoms, belonging to risk group and confirmed diagnosis), health status of close relative or friends (know someone who was diagnosed, COVID-19 outcome), COVID-19-related financial worries and perceived risk of serious illness associated with COVID-19 infection. Participants were asked to answer in a Yes/No format to each of the three following single-item questions on COVID-19-related experiences: (a) ‘Do you belong to an at-risk group for COVID-19 infection?’, (b) ‘Have you been officially diagnosed with COVID-19?’, (c) ‘Have any of your family or friends been seriously ill or passed away due to COVID-19?’. Participants were also asked to answer the following items on 5-point Likert scale (from 1 for ‘Not at all’ to 5 for ‘Extremely’): (d) ‘Do you worry about getting into financial difficulties due to the Corona crisis?’. (e) ‘If someone you know did become infected with the Corona virus, to what extent are you concerned that they will be severely ill?’

### Data Analysis

**Figure 1:**
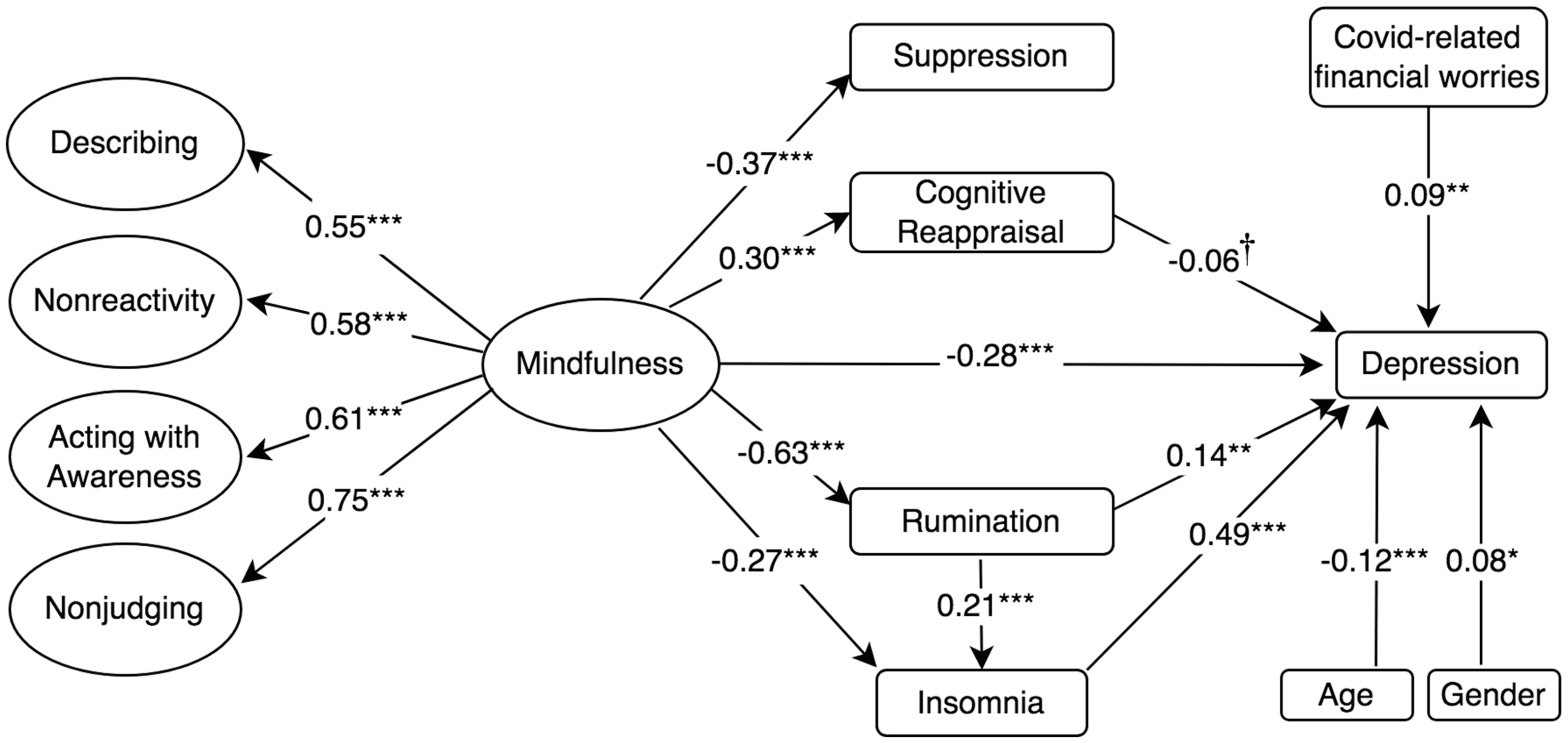
Standardised mediation Model 1 of the effect of higher-order mindfulness on depression. Depression = PHQ-8, 8-item Patient Health Questionnaire; Rumination = RSS-SF, 10-item Rumination Response Scale-Short Form; Cognitive Reappraisal and Suppression = ERQ, 10-item Emotion Regulation Questionnaire; Insomnia = ISI, 7-item Insomnia Severity Index; and Mindfulness and facets = FFMQ-SF, 24-item Five-Facets Mindfulness Questionnaire-Short Form. ^†^*p* < 0.10; ^*^p < 0.05; ^**^p < 0.01; and ^***^p ≤ 0.001.

**Figure 2:**
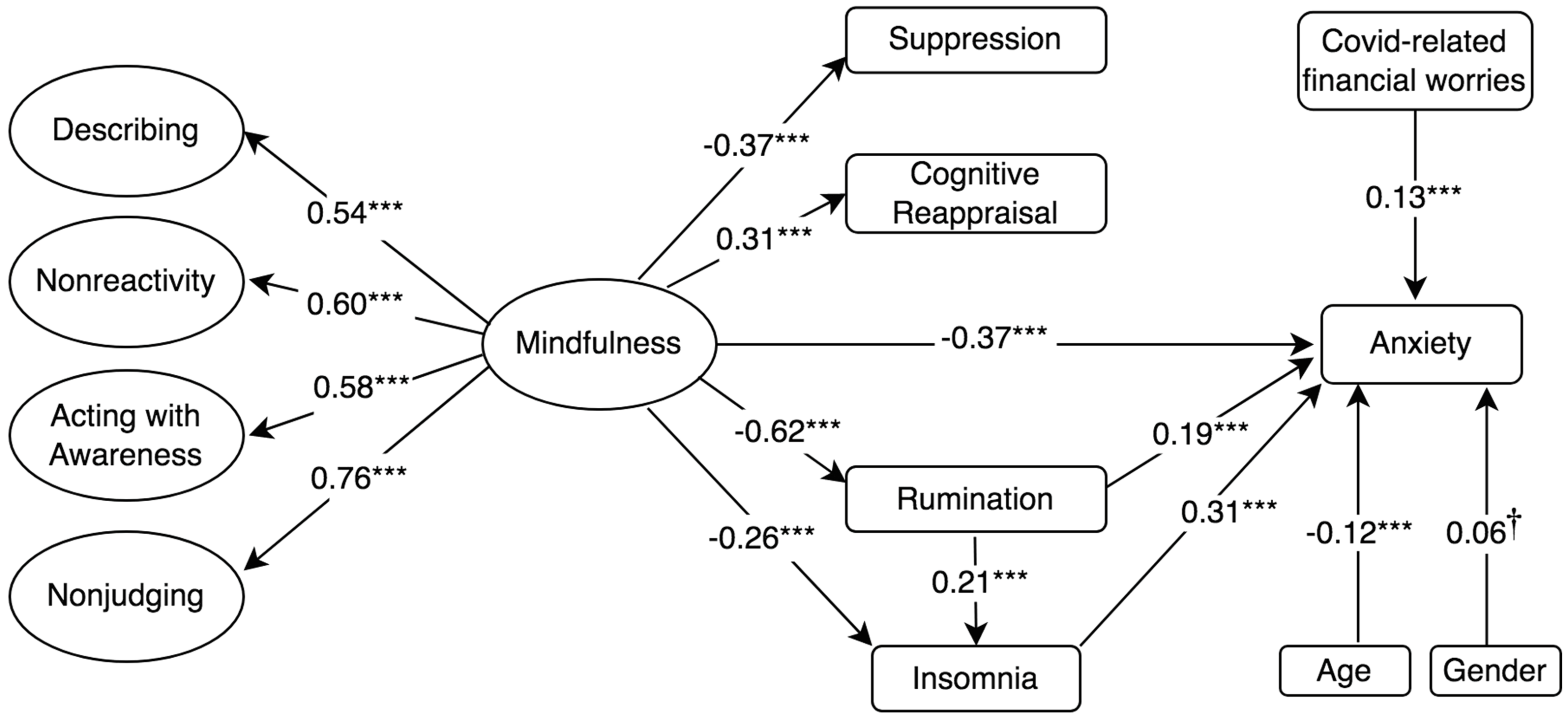
Standardised mediation Model 2 of the effect of higher-order mindfulness on anxiety. Anxiety = GAD-7, a 7-item Generalised Anxiety Disorder; Rumination = RSS-SF, 10-item Rumination Response Scale-Short Form; Cognitive Reappraisal and Suppression = ERQ, 10-item Emotion Regulation Questionnaire; Insomnia = ISI, 7-item Insomnia Severity Index; and Mindfulness and facets = FFMQ-SF, 24-item Five-Facets Mindfulness Questionnaire-Short Form. ^†^*p* < 0.10; ^*^p < 0.05; ^**^p < 0.01; and ^***^p ≤ 0.001.

**Figure 3:**
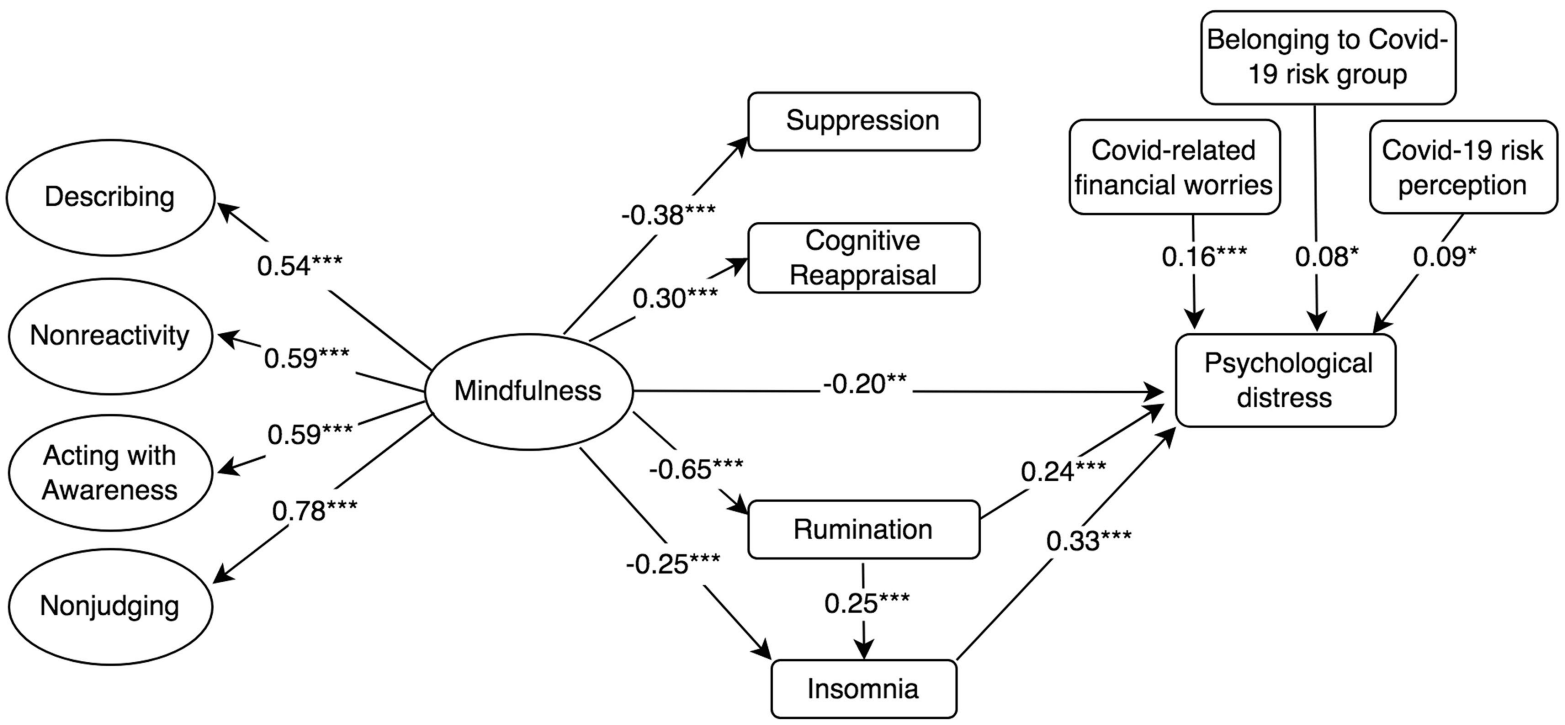
Standardised mediation Model 3 of the effect of higher-order mindfulness on COVID-19-related psychological distress. Psychological impact of COVID-19 = IES-R, 22-item Impact of Event Scale-Revised; Rumination = RSS-SF, 10-item Rumination Response Scale-Short Form; Cognitive Reappraisal and Suppression = ERQ, 10-item Emotion Regulation Questionnaire; Insomnia = ISI, 7-item Insomnia Severity Index; and Mindfulness and facets = FFMQ-SF, 24-item Five-Facets Mindfulness Questionnaire-Short Form. ^†^*p* < 0.10; ^*^p < 0.05; ^**^p < 0.01; and ^***^p ≤ 0.001.

As described in the pre-registration of this study (Mamede et al., 2020), structural equation modelling was used to examine the role of emotion regulation strategies and insomnia on the relationship between mindfulness and mental health problems (symptoms of depression, anxiety and psychological distress). All analyses were performed using the *lavaan* package in R software. A two-step SEM procedure was used to analyse the mediating roles. First, the measurement model of the FFMQ-SF was examined. Subsequently, a path model was used to analyse the fit between the proposed theoretical models (See Figures 1–3 of pre-registration), and the data in our sample. Analysis were conducted using the maximal likelihood estimation, and model fits will be evaluated using multiple indicators, including the chi-squared goodness-of-fit test, the root mean square error of approximation (RMSEA), standardised root mean square residual (SRMR) and the comparative fit index (CFI). We interpreted our findings by following the widely used cut-offs proposed by Hu and Bentler (1999). The RMSEA values of 0.08 or less indicate an acceptable fit, and values of 0.06 or less indicate a good fit. For the SRMR, a value of 0.08 or less indicates good fit. For the CFI, values of 0.90 or higher indicate acceptable fit, and values of 0.95 or higher indicate excellent fit. Furthermore, the Akaike’s Information Criterion (AIC) and the Bayesian Information Criteria (BIC) will also be used to compare models, with lower AIC and BIC values indicating better model fit (West et al., 2012).

#### Factor Structure of FFMQ-SF

The factor structure of the FFMQ-SF will be assessed before investigating the mechanisms underlying the relationships between mindfulness and mental health outcomes. This is necessary because there has been conflicting findings in the literature about the factor structure of the FFMQ, particularly regarding the *observing* facet (Baer et al., 2006; Lilja et al., 2011; Abujaradeh et al., 2019). Since the short form of the FFMQ was used, item parcels would consist of only two or three items. After examining the covariance structure of the data, we opted to not use item parcelling in order to avoid the risk of mis-specifying our model (Little et al., 2009). Based on the original studies developing and validating the FFMQ (Baer et al., 2006, 2008), three different confirmatory factor analysis (CFAs) models were tested and compared for the FFMQ-SF. Firstly, we tested whether the FFMQ-SF measures a unidimensional construct of mindfulness by specifying a model in which all item load on a single factor, which is expected to fit the data poorly (Baer et al., 2006; Bohlmeijer et al., 2011). Subsequently, we examined two five-factor models, a hierarchical five-factor model, which assumes that the five facets are elements of an overall higher-order mindfulness construct and are allowed to correlate, and a non-hierarchical correlated five-factor model, which tests whether the FFMQ-SF scale measures five distinct but related facets of mindfulness. Both five-factor models demonstrated acceptable fit in studies evaluating the psychometric properties of the original FFMQ (Baer et al., 2006) and its short-form version (Bohlmeijer et al., 2011).

In accordance with evidence from the original validation studies of the FFMQ (Baer et al., 2006), a unidimensional single-factor model for the FFMQ-SF showed poor fit to the data, *χ*^2^ (*df* = 252, *N* = 536) = 2627.495, CFI = 0.450, SRMR = 0.124, RMSEA (90% confidence interval [CI]) = 0.133 (0.128, 0.137). This finding suggests that the combination of all the items in the FFMQ-SF do not measure a unidimensional construct of mindfulness. In line with previous studies investigating the factor structure of the FFMQ (Baer et al., 2006) and its short-form version (Bohlmeijer et al., 2011), both five-factor models demonstrated an acceptable fit. The non-hierarchical five-factor model of the FFMQ, *χ*^2^ (*df* = 242, *N* = 493) = 541.2, CFI = 0.926, SRMR = 0.065, RMSEA (90% [CI]) = 0.050 (0.044, 0.056), AIC = 29305.1, BIC = 29548.7, performed slightly better than the hierarchical five-factor model, *χ*^2^ (*df* = 247, *N* = 493) = 567.44, CFI = 0.913, SRMR =0.070, RMSEA (90% [CI]) = 0.051 (0.046, 0.057), AIC = 29321.3, BIC = 29543.9. However, we found that while most FFMQ-SF facets correlated significantly with each other and loaded well into the higher construct of mindfulness, with *r* ranging from 0.32 to 0.75, the *observing* facet was not correlated with *nonreactivity* and *nonjudging* facets and only weakly correlated with the *describing* and *acting with awareness* facets. Additionally, the *observing* facet did not load well into the higher-order mindfulness facet (See Supplementary Figures 7–12 and Supplementary Table 3 in the additional material 1). Removing the *observing* facet significantly improved the fit of both models (*p* ≤ 0.001), with the non-hierarchical four-factor model of the FFMQ-SF, *χ*^2^ (*df* = 164, *N* = 493) = 407.1, CFI = 0.932, SRMR = 0.069, RMSEA (90% confidence interval [CI]) = 0.055 (0.048, 0.062), AIC = 24098.9, BIC = 24292.1, performing just marginally better than the hierarchical four-factor model, *χ*^2^ (*df* = 166, *N* = 493) = 414.65, CFI = 0.930, SRMR = 0.071, RMSEA (90% confidence interval [CI]) = 0.055 (0.049, 0.062), AIC = 24102.5, BIC = 24287.3. Based on these findings and on the previous literature providing support for a four-factor model of mindfulness without the observing subscale (Abujaradeh et al., 2019), we fit our structural equation models testing mediation effects using two different four-factor structures of the FFMQ-SF as independent variables, namely, a hierarchical four-factor model and a non-hierarchical four-factor model of mindfulness.

#### Structural Equation Modelling of Mindfulness Mechanisms

**Figure 4:**
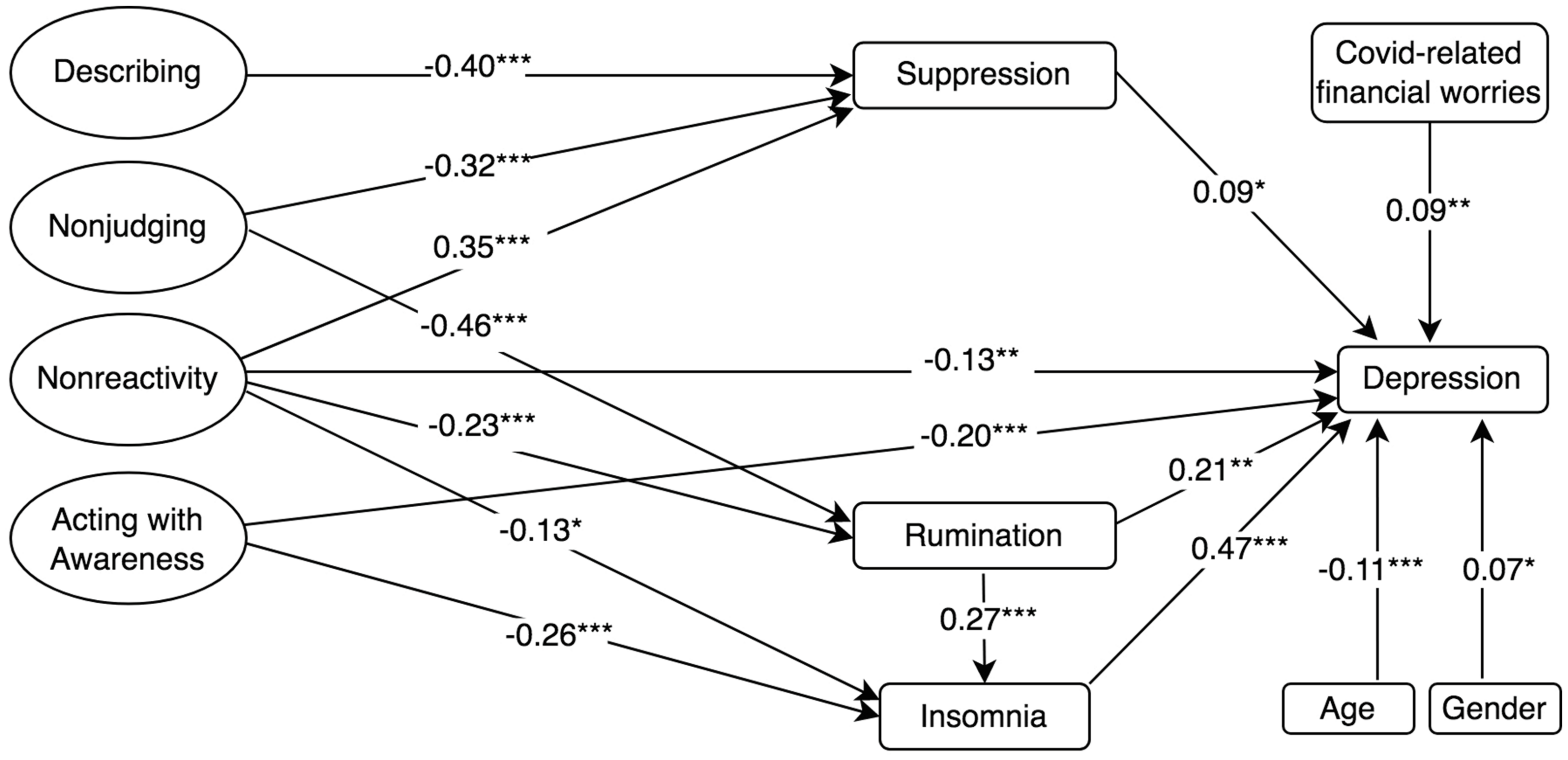
Standardised mediation Model 4 of the effect of the five facets of mindfulness on depression. Depression = PHQ-8, 8-item Patient Health Questionnaire; Rumination = RSS-SF, 10-item Rumination Response Scale-Short Form; Cognitive Reappraisal and Suppression = ERQ, 10-item Emotion Regulation Questionnaire; Insomnia = ISI, 7-item Insomnia Severity Index; and Mindfulness and facets = FFMQ-SF, 24-item Five-Facets Mindfulness Questionnaire-Short Form. ^†^*p* < 0.10; ^*^p < 0.05; ^**^p < 0.01; and ^***^p ≤ 0.001.

In this study, mindfulness was regarded as a latent variable, emotion regulation strategies and sleep problems were considered mediating variables, and symptoms of depression, anxiety and psychological distress were observed outcome variables in Models 1, 2, and 3, respectively. SEM was used to analyse the fit between the proposed theoretical model (See Figures 2–4 of the pre-registration; Mamede et al., 2020) and the sample data. For each outcome variable, two models will be specified based on different factor structures of the FFMQ-SF, one utilising a higher-order construct of mindfulness as the independent variable (Models 1, 2, and 3) and another utilising the different facets of mindfulness as independent variables (Models 4, 5, and 6). As mentioned, a four-factor structure of the FFMQ (excluding the *observing* subscale) was used instead of the five-factor structured described in the pre-registration. Covariates that were not significantly (*p* ≤ 0.10) associated with our outcome variables were excluded from the final models. The analysis of the present paper is described in detail in the pre-registration of this study (Mamede et al., 2020).

## Results

### Descriptive Statistics

As described in the pre-registration, data screening involved the exclusion of responses with extremely short duration and incomplete responses, after which a total of 493 participants were included in the analysis. Table 1 presents sample characteristics, and rates of participants exceeding cut-off for moderate depression, anxiety and insomnia, as well as for COVID-19-related psychological distress, financial worries and risk perception. Regarding nationality, 30.6% of participants were Dutch, 27.0% were German, 7.1% were Australian and 5.9% were British. At the time of data collection, 49.7% of participants resided in Netherlands and 24.2% in Germany. A considerable number of participants in this sample scored above the cut-off for moderate depression (28.8%), anxiety (23.2%), psychological distress (33.8%) and insomnia (19.5%). Additionally, 39.5% of participants indicated experiencing at least moderate COVID-19-related financial worries, and 85.7% of participants responded that they would be at least moderately concerned that they would become seriously ill if they became infected with COVID-19, while 12.2% indicated that they would be extremely concerned.

**Table 1:** Sample characteristics and rates of participants exceeding cut-off for moderate depression, anxiety and insomnia and for COVID-19-related psychological distress, financial worries and risk perception.

Participants (*n* = 493)	
Gender	*n* (%)
Male	152 (30.8%)
Female	334 (68.7%)
Education	*n* (%)
High-school degree or below	254 (51.5%)
Bachelor’s degree	186 (37.7%)
Master’s degree or higher	53 (10.7%)
Age (SD)	25.8 (10.5)
Country of residence	%
Netherlands	49.7%
Germany	24.2%
United Kingdom	4.07%
Others	22.04%
Depression (PHQ-8 score > 10) *n* (%)	141 (28.8%)
Anxiety (GAD-7 score > 10) *n* (%)	113 (23.2%)
Psychological distress (IES-R score > 33) *n* (%)	159 (33.8%)
Insomnia (ISI score > 15) *n* (%)	95 (19.5%)
Covid-related financial worried (>Moderate) *n* (%)	190 (39.5%)
Covid risk perception (>Moderate) *n* (%)	420 (85.7%)
PHQ-8 score (SD)	7.1 (5.3)
GAD-7 score (SD)	6.3 (4.8)
IES-R score (SD)	26.8 (21.9)
ISI score (SD)	8.55 (6.3)

### Structural Equation Modelling of Mindfulness Mechanisms

#### Higher-Order Mindfulness Model

The model assessing the proposed structural relationships between a higher-order construct of mindfulness, the mediators and depressive symptoms (Model 1 in Figure 1) demonstrated an acceptable fit to the data according to some indicators of fit (i.e., RMSEA and *χ*^2^*/df*), but not others, *χ*^2^ (*df* = 338, *N* = 454) = 1080.5, CFI = 0.835, SRMR = 0.098, RMSEA (90% confidence interval [CI]) = 0.070 (0.065, 0.074). In a similar fashion, Models 2 and 3 (Figures 2, 3) with anxiety and psychological distress as outcome variables, respectively, demonstrated acceptable fit to the data according to certain indicators, for example RMSEA, but not according to others, such as the CFI (See Table 2 for comparison of fit indices between models).

**Table 2:** Fit indices among the four-factor models tested.

	IV	DV	*χ* ^2^	*df*	*χ*^2^/*df*	RMSEA	SRMR	CFI	BIC	AIC
Model 1	Higher order	Depression	1080.5	338	2.95	0.07	0.098	0.835	36000.5	35745.2
Model 2	Higher order	Anxiety	1065.6	338	2.92	0.069	0.097	0.824	35912.3	36167.9
Model 3	Higher order	Distress	1024.1	338	2.80	0.066	0.090	0.844	37888.6	38145.2
Model 4	Four facets	Depression	826.1	316	2.38	0.060	0.085	0.887	35534.9	35880.8
Model 5	Four facets	Anxiety	729.3	292	2.27	0.057	0.079	0.901	36243.1	36586.5
Model 6	Four facets	Distress	767.8	316	2.24	0.056	0.077	0.897	37676.3	38023.8

The structural models were then used to test whether emotion regulation strategies and insomnia mediated the relationship between the higher-order construct of mindfulness and mental health outcome variables. Model 1 (Figure 1) showed that mindfulness had a negative direct effect on depression (*β* = −0.28, *p* ≤ 0.001). Mindfulness was also found to have a negative direct effect on rumination (*β* = −0.63, *p* ≤ 0.001) and insomnia (*β* = −0.27, *p* ≤ 0.001). Rumination (*β* = 0.14, *p* = 0.002) and insomnia (*β* = 0.49, *p* ≤ 0.001) were associated with depression. Rumination also had a positive direct effect on insomnia (*β* = 0.21, *p* ≤ 0.001) and partially mediated the relationship between mindfulness and insomnia. In line with our Hypothesis, Model 1 revealed that the negative association between mindfulness and depression was mediated by its negative association with rumination and that the association between rumination and depression was in turn mediated by the positive association between rumination and insomnia, indicating a mediated mediation (*β* = −0.06, *p* = 0.002).

Model 1 also showed that while mindfulness had a positive effect on cognitive reappraisal (*β* = 0.30, *p* ≤ 0.001) and a negative effect on suppression (*β* = −0.37, *p* ≤ 0.001), neither cognitive reappraisal (*β* = −0.06, *p* = 0.06) or suppression (*β* = 0.02, *p* = 0.57) had a significant effect on depression (*p* ≤ 0.05). Therefore, the indirect effects of mindfulness on depression *via* suppression (*β* = −0.01, *p* = 0.57) and cognitive reappraisal (*β* = −0.02, *p* = 0.06) were not significant, although the indirect effect *via* cognitive reappraisal was approaching significance (*p* = 0.06). Model 1 controlled for the negative association between depression and age, as well as for the positive association between female gender, COVID-19-related financial worries and depression. Model 1 accounted for 61.7% of the variance in depressive symptoms, as well as 39.1% in rumination scores, 9% in cognitive reappraisal, 13.8% in suppression, and 18.7% in insomnia.

Mindfulness also had a negative direct effect on anxiety (*β* = −0.37, *p* ≤ 0.001; Model 2). Rumination (*β* = 0.19, *p* ≤ 0.001) and insomnia (*β* = 0.31, *p* ≤ 0.001) had a positive direct effect on anxiety. Model 2 (Figure 2) revealed similar pattern as model 4, wherein mindfulness had a negative indirect effect on anxiety through its negative associations with rumination and insomnia, in a mediated mediation (*β* = −0.04, *p* = 0.003). Models 2 found no indirect effect of mindfulness on anxiety through cognitive reappraisal or suppression. Regarding covariates, the model accounted for the negative association between anxiety and age, as well as for the positive association between female gender, COVID-19-related financial worries and anxiety. Model 2 accounted for 51.7% of the variance in anxiety symptoms.

Model 3 (Figure 3) showed that mindfulness had a negative direct effect on psychological distress (*β* = −0.20, *p* = 0.006; Model 3). Rumination (*β* = 0.24, *p* ≤ 0.001) and insomnia (*β* = 0.33, *p* ≤ 0.001) also had a positive effect on psychological distress. In the same fashion as models 1 and 2, model 3 revealed that mindfulness had a negative indirect effect on psychological distress through its negative association with rumination and insomnia, in a mediated mediation (*β* = −0.05, *p* ≤ 0.001). Model 3 did not reveal any indirect effects of mindfulness on psychological distress through cognitive reappraisal or suppression. Model 3 controlled for positive associations between COVID-19-related psychological distress and COVID-19-related financial worries, risk perception and belonging to a COVID-19 risk group, and accounted for 41.8% of the variance in COVID-19-related psychological distress.

#### Four Facets of Mindfulness Model

**Figure 5:**
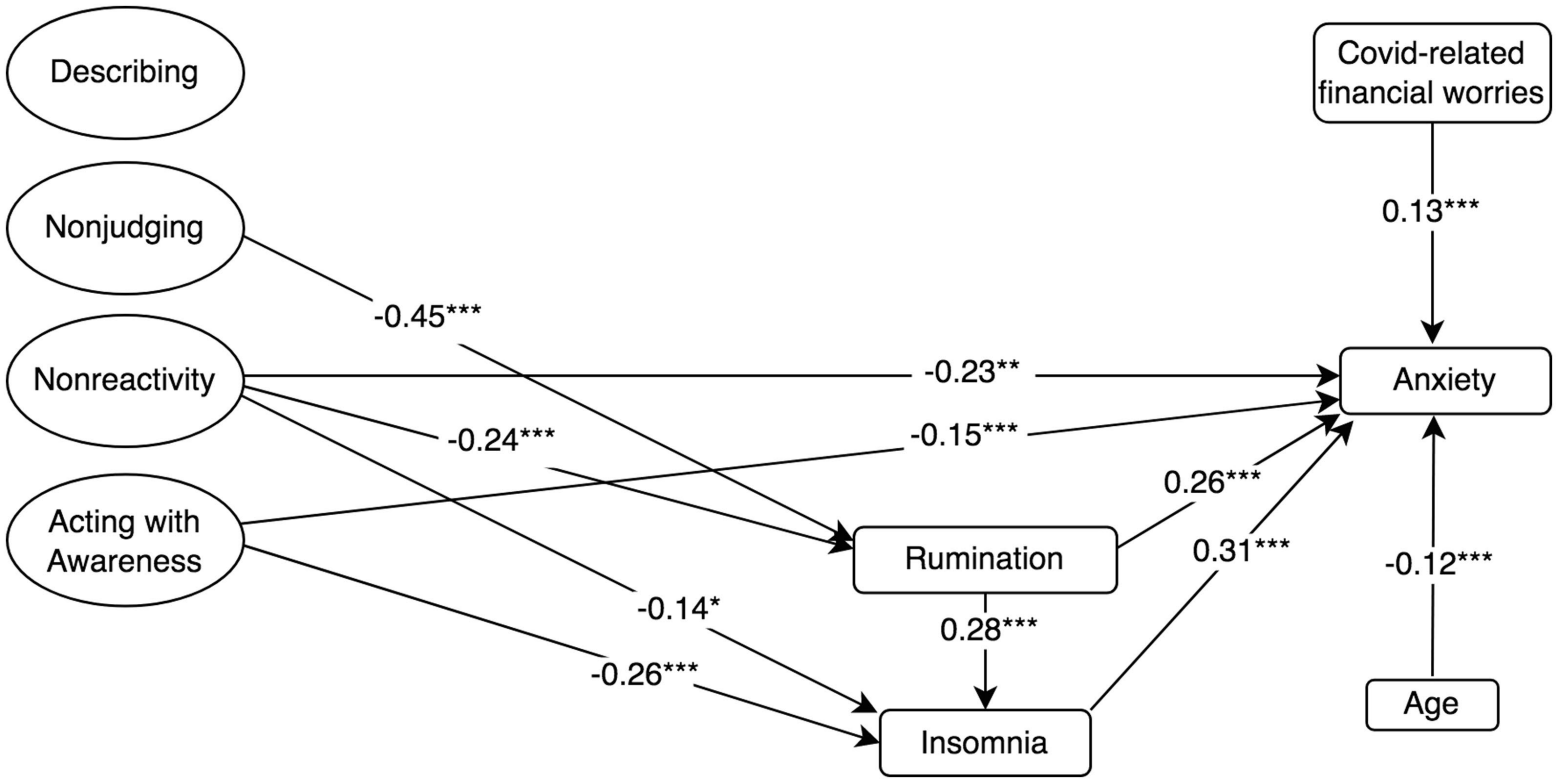
Standardised mediation Model 5 of the effect of the five facets of mindfulness on anxiety. Anxiety = GAD-7, a 7-item Generalised Anxiety Disorder; Rumination = RSS-SF, 10-item Rumination Response Scale-Short Form; Cognitive Reappraisal and Suppression = ERQ, 10-item Emotion Regulation Questionnaire; Insomnia = ISI, 7-item Insomnia Severity Index; and Mindfulness and facets = FFMQ-SF, 24-item Five-Facets Mindfulness Questionnaire-Short Form. ^†^*p* < 0.10; ^*^p < 0.05; ^**^p < 0.01; and ^***^p ≤ 0.001.

**Figure 6:**
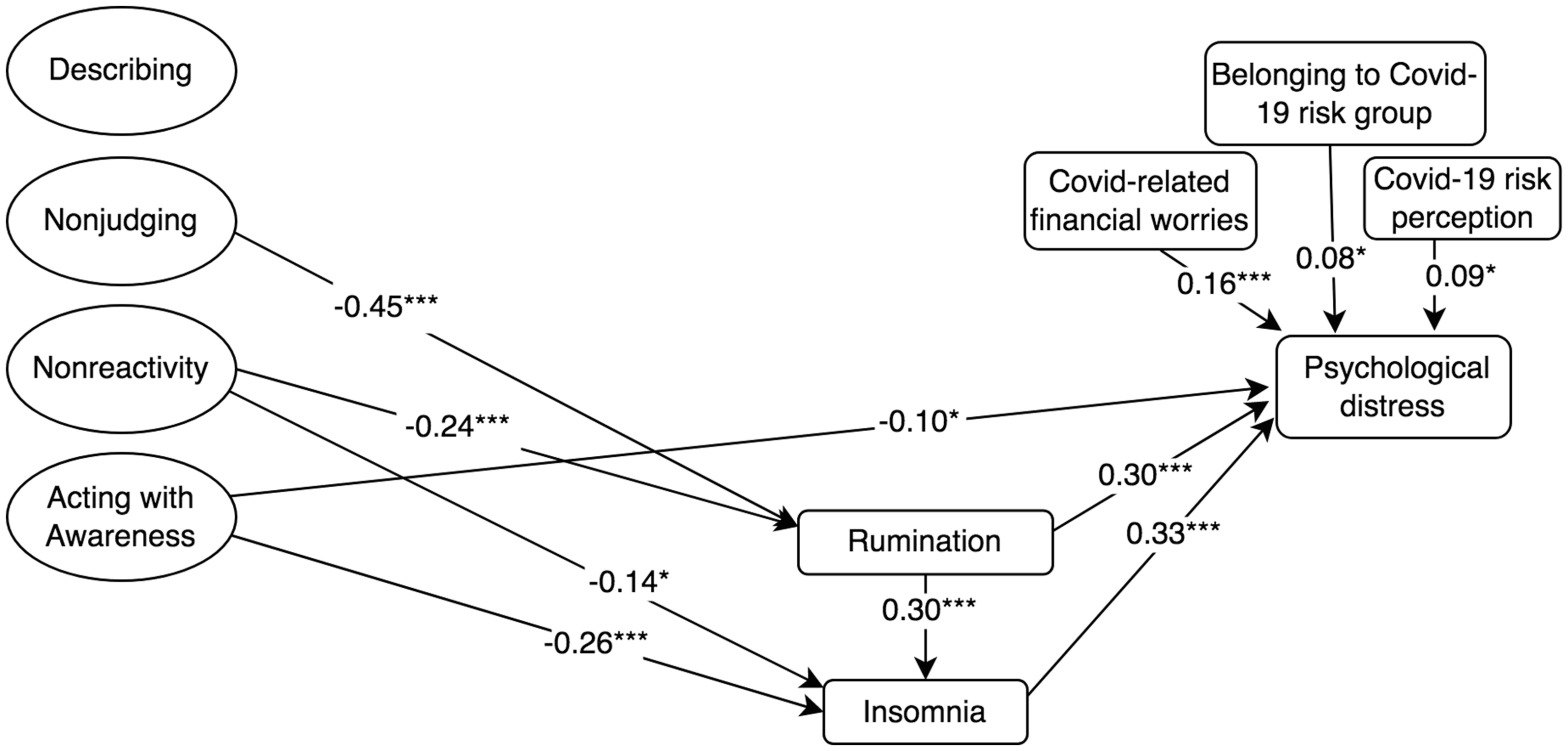
Standardised mediation Model 6 of the effect of the five facets of mindfulness on COVID-19-related psychological distress. Psychological impact of COVID-19 = IES-R, 22-item Impact of Event Scale-Revised; Rumination = RSS-SF, 10-item Rumination Response Scale-Short Form; Cognitive Reappraisal and Suppression = ERQ, 10-item Emotion Regulation Questionnaire; Insomnia = ISI, 7-item Insomnia Severity Index; and Mindfulness and facets = FFMQ-SF, 24-item Five-Facets Mindfulness Questionnaire-Short Form. ^†^*p* < 0.10; ^*^p < 0.05; ^**^p < 0.01; and ^***^p ≤ 0.001.

The models assessing the proposed structural relationships between the four facets of mindfulness, the mediators and depressive symptoms (Model 4 in Figure 4) demonstrated a good fit according to two fit indices (i.e., RMSEA and *χ*^2^*/df*), but not according to another (i.e., CFI), *χ*^2^ (*df* = 316, *N* = 493) = 826.1, CFI = 0.887, SRMR = 0.085, RMSEA (90% [CI]) = 0.060 (0.055, 0.065). This pattern was similar for Models 5 and 6 that included, respectively, anxiety and psychological distress symptoms as outcome variables. Model 5 demonstrated a good model fit according to all indices and Model 6 demonstrated a good fit to the data according to three indices (i.e., RMSEA, SRMR and *χ*^2^*/df*), but not according to another (i.e., CFI; See Table 2). Our findings indicated that, compared to the hierarchical four-factor model, the correlated four-factor model of mindfulness significantly improved the fit of the proposed theoretical models investigating the relationship between mindfulness and depression (*χ*^2^_diff_ = 254.3, *p* ≤ 0.001), anxiety (*χ*^2^_diff_ = 336.3, *p* ≤ 0.001) and psychological distress (*χ*^2^_diff_ = 256.4, *p* ≤ 0.001). These structural models were used to test whether emotion regulation strategies (rumination, cognitive reappraisal and suppression) and sleep problems mediated the relationship between the facets of mindfulness and symptoms of depression (Figure 4), anxiety (Figure 5) and psychological distress (Figure 6).

Mindfulness facets *nonreactivity* and *acting with awareness* had a negative direct effect on depression (Model 4; Figure 4). *Nonjudging* and *nonreactivity* facets were negatively associated with rumination, whereas insomnia was negatively associated with *acting with awareness* and *nonreactivity*. Rumination (*β* = 0.21, *p* ≤ 0.001) and insomnia (*β* = 0.47, *p* ≤ 0.001) were positively associated with depression. Rumination had a positive direct effect on insomnia (*β* = 0.27, *p* ≤ 0.001) and mediated the negative relationship between the *nonjudging* and *nonreactivity* facets and insomnia. Model 4 revealed that rumination mediated the negative association between *nonjudging*, *nonreactivity* and depression, as well as that the positive association between rumination and depression was, in turn, mediated by insomnia, indicating mediated mediation (Figure 4). Suppression was negatively associated with *describing* and *nonjudging* but was positively associated with *nonreactivity.* Suppression was positively associated with depression (*β* = 0.09, *p* < 0.05) and that suppression mediated the negative indirect effects of *describing* (*β* = −0.03, *p* < 0.05), *nonjudging* (*β* = −0.03, *p* < 0.05) and the positive indirect effect of *nonreactivity* (*β* = 0.03, *p* < 0.05) on depression. Cognitive reappraisal was positively associated with the *acting with awareness* (*β* = 0.15, *p* < 0.01), *nonreactivity* (*β* = 0.45, *p* ≤ 0.001) and *describing* (*β* = 0.24, *p* ≤ 0.001) facets, but negatively associated with *nonjudging* (*β* = −0.3, *p* ≤ 0.001). However, cognitive reappraisal was not negatively associated with depression. Finally, Model 4 also controlled for the negative association between age and depression, as well as for the positive associations between depression, female gender and COVID-19-related financial worries. Model 4 accounted for 61.8% of the variance in depressive symptoms, as well as 37.8% in rumination scores, 28.8% in cognitive reappraisal, 33.6% in suppression and 21.4% in insomnia.

Model 5 demonstrated that the *nonreactivity* and *acting with awareness* facets also had a negative direct effect on anxiety. Regarding rumination and insomnia, Model 5 demonstrated a similar pattern as Model 4, wherein mindfulness facets *nonreactivity* and *nonjudging* had negative indirect effect on anxiety through rumination and insomnia, in a mediated mediation (See Figure 5). However, Model 5 did not reveal any effect of suppression or cognitive reappraisal on anxiety. Model 5 controlled for the positive and negative associations between anxiety and the covariates financial worries and age, respectively, and accounted for 52.5% of the variance in symptoms of anxiety.

Model 6 showed that only the *acting with awareness* facet had a negative direct effect on COVID-19-related psychological distress. Model 6 also showed that the *nonreactivity* and *nonjudging* facets had a negative indirect effect on psychological distress through rumination and insomnia, in a mediated mediation (See Figure 6). There were no effects of suppression or cognitive reappraisal on psychological distress. The model controlled for the positive associations between psychological distress and the covariates COVID-19-related financial worries, risk perception and belonging to a COVID-19 risk group. Model 6 accounted for 41.6% of the variance COVID-19-related psychological distress.

### Additional Analysis

The *observing* facet was removed from our models because it loaded poorly into the overall mindfulness facet and did not correlate with other facets of mindfulness. Nonetheless, given the conflicting findings in the literature regarding the observing facet, we also fit the standard mediation models using the hierarchical and non-hierarchical five-factor structure to examine the associations with the observing facet. The model fit indices and the standardised mediation models of the hierarchical and non-hierarchical five-factor models are presented in Supplementary Table 4 in the additional material. Most notably, our findings revealed that the *observing* facet was positively associated with mental health problems and rumination, which was the opposite compared to other mindfulness facets.

## Discussion

The present study sheds light on the potential mechanisms through which mindfulness can influence mental health by demonstrating that certain emotion regulation strategies (i.e., rumination, cognitive reappraisal and suppression) and insomnia mediate the relationship between mindfulness and symptoms of depression, anxiety and psychological distress. Besides exploring the factor structure of the FFMQ-SF, this study investigated whether different facets of mindfulness and the mediators selected operate transdiagnostically or specifically for different psychopathologies. The present study demonstrated that, differently from cognitive reappraisal and suppression, rumination operated transdiagnostically as a mediator of the effects of mindfulness on symptoms of depression, anxiety and psychological distress during COVID-19. In line with our expectations, the relationships between higher trait mindfulness and mental health outcomes were also mediated by reductions in insomnia symptoms, which, in turn, were mediated by lower levels of rumination. This study also found support for a four-factor structure of the FFMQ-SF (i.e., excluding the *observing* facet) and demonstrated both specific and transdiagnostic effects of certain facets of mindfulness on symptoms of depression, anxiety and psychological distress.

## Mindfulness and Mental Health: Emotion Regulation and Insomnia

In our sample, higher-order mindfulness was directly and negatively associated with all mental health problems. Regarding mindfulness facets, *acting with awareness* and *nonreactivity* were directly linked to anxiety and depression, but only *acting with awareness* was directly related to psychological distress. As hypothesised, all of our models revealed that the association between mindfulness and mental health outcomes was significantly mediated by its negative association with rumination. This result is in line with previous studies demonstrating that rumination mediates the effects of mindfulness on mental health (Desrosiers et al., 2013; Parmentier et al., 2019) and indicates that rumination operates transdiagnostically as a mechanism of the protective effects of mindfulness on depression, anxiety and psychological distress.

Rumination is characterised by a pattern of repetitive negative thoughts about an emotional experience or situation. Theorists have suggested that the *nonjudging* and *acting with awareness* facets of mindfulness may be particularly protective against rumination, which could in turn explain how mindfulness may reduce symptoms of anxiety and depression (Brown et al., 2007; Nolen-Hoeksema et al., 2008). The *acting with awareness* involves consciously attending to moment-to-moment experience. It is plausible that higher levels of such facet may facilitate early recognition, control and disengagement from repetitive thinking patterns characteristics of rumination. *Nonjudging* involves facing one’s internal experiences with an acceptant attitude rather than a judgemental one. Higher levels of *nonjudging* may lessen engagements with negative evaluative thoughts, as well as facilitate the acceptance of negative thoughts and feelings as transient experiences, which may reduce the initiation and perpetuation of rumination. Our findings partially support this notion, as our models 4, 5 and 6 revealed that the facets *nonjudging* and *nonreactivity* were negatively associated to rumination, which mediated the link between these facets and mental health outcomes, whereas *acting with awareness* was directly associated with mental health outcomes. In contrast with previous research (Petrocchi and Ottaviani, 2016; Iani et al., 2019), *nonreactivity* was also negatively associated with rumination in our models, which is plausible considering that nonreactivity and acceptance to certain moods could prevent the triggering of ruminative responses in face of negative moods or stimuli. Finally, our exploratory analysis revealed that the *observing* facet was positively associated with rumination, although it has been argued that this relationship may depend on the way in which individuals observe their experiences, which may depend on other mindfulness facets or on meditation experience (Desrosiers et al., 2014). Further research is needed to further examine the relationship between facets of mindfulness and rumination.

Our findings also confirmed our hypothesis that mindfulness was negatively associated with insomnia symptoms and that this relationship was mediated by rumination. Insomnia was directly associated with the *acting with awareness* and *nonreactivity* facets of mindfulness, as well as indirectly associated with *nonjudging* and *nonreactivity* through rumination. Several studies have shown that mindfulness has a positive influence on sleep (Rusch et al., 2019), but few studies have examined emotion regulation strategies (e.g., rumination) as possible mediators (Liu et al., 2018; Calvete and Joana, 2021). The evidence from these studies is consistent with our finding that mindfulness, particularly the *nonjudging, nonreactivity* and *acting with awareness* facets, seems to improve sleep and reduces insomnia symptoms either directly or indirectly by reducing rumination.

Furthermore, in our sample, insomnia mediated the associations between mindfulness and depression, anxiety and psychological distress, indicating that sleep may be a transdiagnostic working mechanism of the positive effects of mindfulness on mental health. Therefore, it seems that mindfulness not only affects mental health by reducing rumination, but also that these improvements in mood regulation translate to fewer insomnia symptoms, which in turn also positively influences mental health. These results are supported by both theory and empirical evidence, which suggests that emotion regulation, particularly rumination, plays an crucial role in sleep disturbances (Nolen-Hoeksema et al., 2008; Pillai and Drake, 2015) and that sleep, in turn, has a profound effect on mental health (Hall et al., 2000; Alvaro et al., 2011; Lopresti et al., 2013). Remarkably, while sleep is certainly affected by emotion regulation, evidence demonstrates that sleep can also influence emotional reactivity and the regulation of positive and negative emotions, suggesting a complex interplay between sleep and emotion regulation (Gruber and Cassoff, 2014). Research has found that sleep influences stress hormones and inflammation (Wright et al., 2015), which have been shown to be involved in the development of depression (Iob et al., 2020), anxiety (Donovan et al., 2010) and psychological distress (Goldman-mellor et al., 2012). These pathways may partially explain how the complex interplay between sleep and emotion regulation, primarily rumination, operate transdiagnostically and contribute to different forms of psychopathology. Although further research is needed to explore the complex mechanisms linking sleep, emotion regulation and mental health, the present study demonstrates that mindfulness, particularly its *nonjudging*, *acting with awareness* and *nonreactivity* facets, seems to have a positive influence on mental health through reductions in rumination and insomnia symptoms. Future studies with experimental and longitudinal designs are needed to confirm our findings and further examine how increasing mindfulness and its facets can possibly prevent and treat mental health problems through improvements in emotion regulation and sleep behaviour. By using these insights, future research can develop more effective mindfulness interventions for mental health by, for instance, focusing on *nonjudging*, *nonreactivity* and *acting with awareness* facets of mindfulness, which seem to reduce sleep problems both directly and through reductions in rumination.

Our hypothesis on cognitive reappraisal and suppression as working mechanisms of mindfulness were only partially supported, as our findings indicated that cognitive reappraisal and suppression may mediate the effects of mindfulness on depression, but not on anxiety or psychological distress. Therefore, contrary to our expectations, cognitive reappraisal and suppression did not operate as transdiagnostic mediators of mindfulness, but rather specifically for depression. Cognitive reappraisal seemed to specifically mediate the influence of a higher-order mindfulness construct on depression, although this did not reach statistical significance. This result is in line with previous research demonstrating that cognitive reappraisal mediated the effects of mindfulness on depression, but not anxiety (Desrosiers et al., 2013), as well as with neuroimaging research indicating that dispositional mindfulness is associated with greater activation of brain regions responsible for emotion regulation during a reappraisal task (Modinos and Ormel, 2010). However, our finding is somewhat incongruent with one previous study which showed that cognitive reappraisal mediated the effects of mindfulness on both anxiety and depression (Parmentier et al., 2019). Therefore, further research is needed to examine whether cognitive reappraisal mediates the effects of mindfulness on anxiety. Additionally, our models revealed that suppression did not mediate the influence of higher-order mindfulness on depression, but it was a significant mediator of the effect of certain facets of mindfulness on depression, namely, the *describing*, *nonjudging* and *nonreactivity* facets. These findings are congruent with a previous studies examining the working mechanisms of mindfulness, which found that suppression significantly mediated the effects of mindfulness on depression, but not on anxiety (Parmentier et al., 2019). However, our results differ in that suppression did not significantly mediate the effects of higher-order mindfulness on depression, but rather mediated the effects of the *describing*, *nonjudging* and *nonreactivity* facets. It is important to note that, in line with previous literature (Zhang et al., 2019), we found that *nonreactivity* had a positive association with suppression, whereas *describing* and *nonjudging* had a negative association, which might be the explanation why suppression did not mediate the effect of higher-order mindfulness on depression in Model 1. Although further studies are needed to confirm our findings, future intervention studies can use these insights to target mindfulness facets that are most closely linked to poor emotion regulation, thereby more effectively reducing suppression and rumination. Together with the evidence from previous studies (Desrosiers et al., 2013; Parmentier et al., 2019), our findings suggest that while rumination and sleep operate as common mediating mechanisms of the effects of mindfulness on depression, anxiety and psychological distress, cognitive reappraisal and suppression appear to operate specifically on depression.

## Measuring Mindfulness: Psychometric Properties of the FFMQ-SF

In addition to investigating the working mechanisms of mindfulness, this study explored the psychometric properties of the FFMQ-SF. The confirmatory factor analysis provided the most support for both the hierarchical four-factor model and the non-hierarchical four-factor models of the FFMQ-SF, without the *observing* facet. This finding is in line with the original study investigating the psychometric properties of the FFMQ (Baer et al., 2006), as well as with subsequent studies investigating the validity of the Swedish (Lilja et al., 2011) and short-form English (Abujaradeh et al., 2019) versions of the questionnaire, which demonstrated that the *observing* facet was not a significant part of self-reported mindfulness in populations with limited meditation experience. Remarkably, while most facets of mindfulness were negatively associated with mental health outcomes in our sample, particularly the *acting with awareness*, *nonjudging* and *nonreactivity*, the *observing* facet was consistently positively associated with mental health problems, either directly or indirectly through its positive association with rumination. This is in line with findings from previous studies exploring the relationship between mindfulness facets, rumination and depression in adolescents (Royuela-Colomer and Calvete, 2016), which also found that *acting with awareness* and *nonreactivity* had a positive impact on mental health, whereas the observing facet had the opposite effect. Thus, while our findings provide support for the adaptive role of several facets of mindfulness, such as *acting with awareness* and *nonreactivity*, it seems that the observing facet may play a maladaptive role in the general population.

There are several possible explanations for the inconsistent functioning of the *observing* facet. One study found that *observing* was correlated with psychological adjustment in meditators, but not in nonmeditating samples (Baer et al., 2008). Baer et al. (2008) suggested that while the *observing* facet may capture maladaptive forms of attention in nonmeditators, a higher score on *observing* in meditators may simply reflect a greater tendency to attend to a range of internal and external stimuli, rather than attending selectively to threatening or unpleasant ones. Recent studies have also suggested that the *observing* facet in the FFMQ lacks items assessing awareness of emotion and that this may explain the unexpected relationships found between the *observing* facet, psychological symptoms and other mindfulness facets (Rudkin et al., 2018). For example, this may explain differences between meditators and nonmeditators, since meditation trains the nonevaluative and accepting observation of all stimuli, including emotions, thoughts and external stimuli, the *observing* facet may in fact capture the awareness of emotions for meditators, but not for nonmeditators.

## Limitations and Implications

The current study should be interpreted in light of certain limitations. Firstly, the cross-sectional design limits the interpretation of causality between variables of interest in our models. Future research should conduct experimental or longitudinal studies involving mindfulness interventions to further examine the behavioural and cognitive mechanisms explored in our mediation analysis. Moreover, the collection of the data was based exclusively on self-report measures. To improve the validity of our findings and to further explore the mechanisms linking mindfulness to mental health, future studies could implement additional measurements methods, such as neuroimaging measures and objective assessments of sleep duration and quality. Considering that this study relied on data from an international sample consisting largely of university students, future research is also needed to examine generalisability of our findings to other populations (e.g., low-income older adults). Finally, we did not assess for meditation experiences, which would have allowed us to explore possible differences between meditators and non-mediators.

Despite these constraints, several strengths and implications of the current study should be mentioned. This study showed that rates of depression, anxiety, psychological distress and insomnia were considerably higher during the initial period of the pandemic (May–June 2022) than prevalence rates reported in pre-pandemic studies with international (Ohayon, 2002; Varma et al., 2021) and Dutch samples (De Graaf et al., 2012; Ormel et al., 2015). Additionally, a large proportion of participants reported moderate-to-extreme COVID-19-related financial worries and risk perception. Although some research has observed resilience to mental health problems during the pandemic in certain groups (e.g., older adults; Fields et al., 2021), we observed elevated rates of mental health problems in this study’s international sample consisting mainly of young adults residing in Netherlands and Germany. These findings reinforce the importance of investigating, as we did, whether and how mindfulness could partially explain the heterogeneity in risk for psychopathology during the COVID-19 pandemic.

Our findings contributed to the existing evidence (Desrosiers et al., 2013; Freudenthaler et al., 2017; Parmentier et al., 2019) supporting the role of emotion regulation strategies as mediators of the effects of mindfulness on mental health. This study also added to the literature (Gruber and Cassoff, 2014; Pillai and Drake, 2015) by investigating whether and how the interplay between maladaptive emotion regulation (i.e., rumination) and insomnia mediates the relationship between mindfulness and mental health in a large sample. Our findings indicated that both rumination and sleep operate transdiagnostically as working mechanisms of mindfulness, and our models revealed that the *acting with awareness*, *nonjudging* and *nonreactivity* facets of mindfulness were most strongly related to decreases in rumination and insomnia symptoms. Future MBIs could more effectively improve mental health by targeting mindfulness facets that are more strongly associated with reductions in rumination (i.e., *nonjudging* and *nonreactivity*) and insomnia (i.e.*, acting with awareness*). This study also emphasises the benefit of targeting mindfulness interventions for individuals with poor emotion regulation and sleep. Nonetheless, given the strong associations between mindfulness, mental health and improved emotion regulation and sleep, our results also highlight the potential usefulness of offering MBIs to the general population, particularly in times of crisis, such as the COVID-19 pandemic. Given that mindfulness buffers stress, the effects of mindfulness on mental health observed in this study may have been particularly robust due to the study being conducted during a highly stressful period. However, similar findings have been observed in pre-pandemic studies (Desrosiers et al., 2013; Parmentier et al., 2019). Even though levels of stress and rumination observed in pre-pandemic studies could be expected to be lower, they were nonetheless relevant for mental health. Therefore, we argue that the effects of mindfulness on emotion regulation, sleep and mental health observed in the present study are also likely applicable to normal circumstances.

Moreover, our findings indicated that cognitive reappraisal and suppression were relevant mediators of the effects of mindfulness on depression, but not for anxiety or psychological distress. Although further research is warranted to confirm these findings, especially given the conflicting findings in the literature regarding, for example, the role of cognitive reappraisal in anxiety (Desrosiers et al., 2013; Parmentier et al., 2019), these results indicate that certain emotion regulation strategies like cognitive reappraisal may be particularly relevant for depressive symptoms, rather than operate transdiagnostically for various psychopathologies. Finally, in an exploratory fashion, this study also examined the psychometric properties of the FFMQ-SF and found the greatest support for a four-factor model without the *observing* facet, which is in line with previous literature. Future studies should continue to measure the elements of mindfulness separately in order to investigate how meditation practice may affect them differently, as well as to explore how the facets may relate differently with other variables of interest. These insights may shed light on the specific processes that are influenced by meditation and their role in promoting mental health, which could guide the tailoring of future MBIs to improve their effectiveness.

## Conclusion

Findings of our structural equation models suggest that mindfulness and its facets impact mental health both directly and indirectly through emotion regulation strategies and insomnia. *Acting with awareness*, *nonreactivity* and *nonjudging* seemed to be the mindfulness facets that exerted the strongest positive influence on mental health, emotion regulation and insomnia symptoms. Rumination and sleep seem to be interconnected mediating mechanisms of the effects of mindfulness on symptoms of depression, anxiety and psychological distress, operating transdiagnostically, whereas cognitive reappraisal and suppression functioned specifically as mechanisms for depression and had less robust effects. These findings emphasise the need of disentangling the unique components of mindfulness, emotion regulation and their potential interactions with health behaviours to more clearly understand the mechanisms through which mindfulness can influence symptoms of depression, anxiety and psychological distress. Such insights can facilitate the development of more effective mindfulness interventions for mental health by, for instance, guiding the tailoring and/or targeting of mindfulness interventions for individuals with poor emotion regulation or sleep. Moreover, our findings emphasise the potential benefits of offering mindfulness interventions to the general population in order to prevent and treat mental health problems, particularly in times of crisis such as during the COVID-19 pandemic.

## Data Availability Statement

The raw data supporting the conclusions of this article will be made available by the authors, without undue reservation.

## Ethics Statement

This study was approved by the Ethics Review Committee of the Department of Psychology, Education and Child Studies, Erasmus University Rotterdam (application number 20-051). The patients/participants provided their written informed consent to participate in this study.

## Author Contributions

AM took the lead in the conception and design for the study. AM was responsible for collecting, analysing and interpreting the data, as well as for drafting the manuscript under the supervision and with feedback from IM, GN, and SD. IM, GN, and SD critically revised the manuscript, provided feedback, and contributed to writing the manuscript. All authors contributed to the article and approved the submitted version.

## Funding

This work was supported by Erasmus Open Access fund.

## Conflict of Interest

The authors declare that the research was conducted in the absence of any commercial or financial relationships that could be construed as a potential conflict of interest.

## Publisher’s Note

All claims expressed in this article are solely those of the authors and do not necessarily represent those of their affiliated organizations, or those of the publisher, the editors and the reviewers. Any product that may be evaluated in this article, or claim that may be made by its manufacturer, is not guaranteed or endorsed by the publisher.
