# Supplementary material for: Mindfulness as a Protective Factor Against Depression, Anxiety and Psychological Distress During the COVID-19 Pandemic: Emotion Regulation and Insomnia Symptoms as Mediators
Source: Front Psychol. 2022 Apr 1;13:820959. doi: 10.3389/fpsyg.2022.820959 (PMC9010863; doi:10.3389/fpsyg.2022.820959)
Supplement: Supplementary file 1 [file Table_1.DOCX]

**Additional Material 1**

**Table 3.** Pearson’s correlation between all dependent, independent and mediating variables.

|  | 1 | 2 | 3 | 4 | 5 | 6 | 7 | 8 | 9 | 10 | 11 | 12 | 13 |
| --- | --- | --- | --- | --- | --- | --- | --- | --- | --- | --- | --- | --- | --- |
| 1. Depression | - |  |  |  |  |  |  |  |  |  |  |  |  |
| 1. Anxiety | **0.78** | - |  |  |  |  |  |  |  |  |  |  |  |
| 1. Distress | **0.63** | **0.67** | - |  |  |  |  |  |  |  |  |  |  |
| 1. Rumination | **0.57** | **0.59** | **0.52** | - |  |  |  |  |  |  |  |  |  |
| 1. Suppression | **0.21** | **0.16** | 0.13** | **0.20** | - |  |  |  |  |  |  |  |  |
| 1. Cognitive Reappraisal | **-0.23** | **-0.18** | -0.08 | -0.12† | -0.03 | - |  |  |  |  |  |  |  |
| 1. Sleep Problems | **0.65** | **0.55** | **0.53** | **0.4** | 0.11* | -0.1* | - |  |  |  |  |  |  |
| 1. Mindfulness | **-0.52** | **-0.49** | **-0.36** | **-0.44** | **-0.33** | **0.42** | **-0.32** | - |  |  |  |  |  |
| 1. Observing | 0.06 | 0.08* | 0.11** | **0.18** | -0.02 | **0.21** | 0.08† | **0.38** | - |  |  |  |  |
| 1. Describing | **-0.29** | **-0.24** | **-0.2** | **-0.24** | **-0.42** | **0.32** | -0.15** | **0.66** | 0.09* | - |  |  |  |
| 1. Nonreactivity | **-0.42** | **-0.45** | **-0.29** | **-0.4** | 0.07 | **0.40** | **-0.28** | **0.63** | 0.01 | **0.24** | - |  |  |
| 1. Nonjudging | **-0.40** | **-0.43** | **-0.36** | **-0.53** | **-0.32** | 0.05 | **-0.24** | **0.63** | -0.04 | **0.30** | **0.35** | - |  |
| 1. Acting with awareness | **-0.5** | **-0.44** | **-0.34** | **-0.33** | **-0.28** | **0.27** | **-0.35** | **0.73** | **0.16** | **0.37** | **0.29** | **0.36** | - |

*Note:* Boldface = *p* < 0.001; *p* <0.01**; *p* <0.05*; *p* <0.1 †

**Table 4.** Fit indices among the five-factor models tested

|  | IV | DV | χ2 | df | χ2/df | RMSEA | SRMR | CFI | BIC | AIC |
| --- | --- | --- | --- | --- | --- | --- | --- | --- | --- | --- |
| Model 7 | Hierarchical | Depression | 1330.4 | 451 | 2.95 | 0.066 | 0.094 | 0.825 | 40807.6 | 40515.2 |
| Model 8 | Hierarchical | Anxiety | 1318.6 | 451 | 2.92 | 0.065 | 0.095 | 0.824 | 40993.9 | 40701.2 |
| Model 9 | Hierarchical | Distress | 1264.6 | 451 | 2.80 | 0.062 | 0.088 | 0.834 | 43086.29 | 42792.51 |
| Model 10 | Non-hierarchical | Depression | 1003.5 | 421 | 2.38 | 0.055 | 0.079 | 0.884 | 40664.16 | 40248.24 |
| Model 11 | Non-hierarchical | Anxiety | 957.6 | 421 | 2.27 | 0.052 | 0.073 | 0.892 | 41492.63 | 41074.72 |
| Model 12 | Non-hierarchical | Distress | 941.9 | 421 | 2.24 | 0.052 | 0.072 | 0.894 | 42947.74 | 42529.83 |


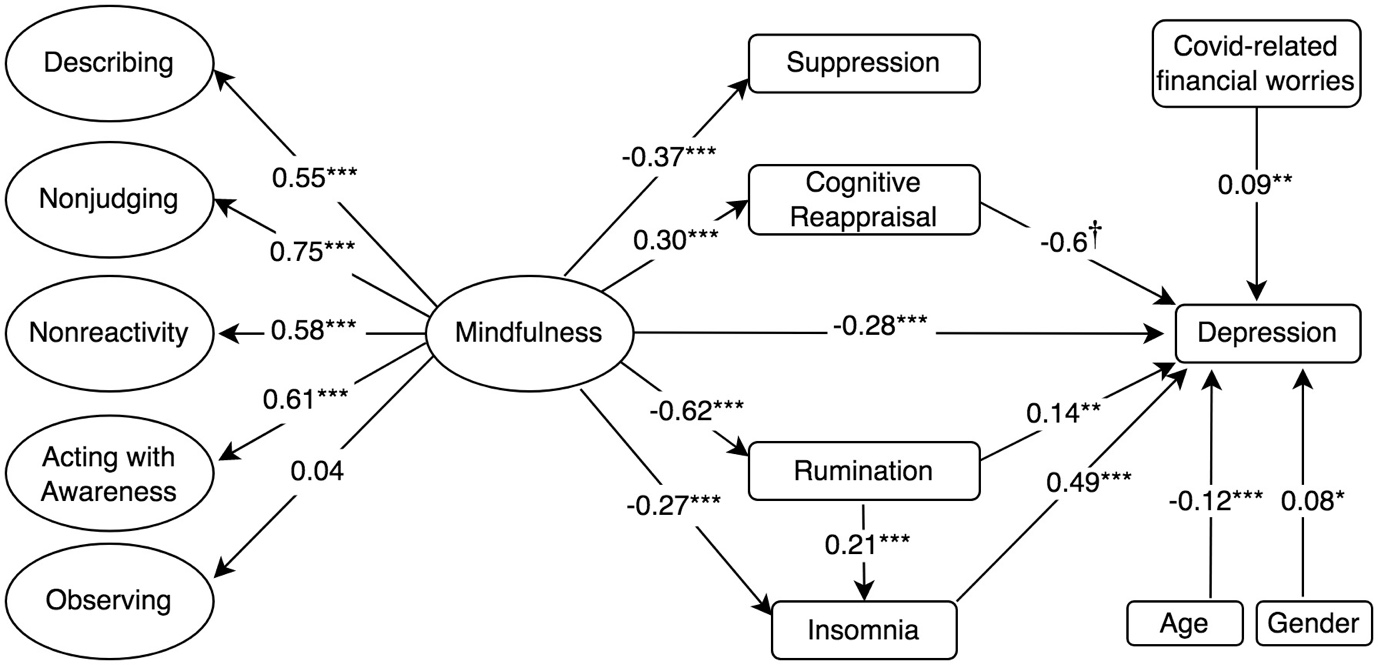


**Fig 7.** Standardized mediation Model 7 of the effect of higher-order mindfulness on depression. Depression = PHQ-8, 8-item Patient Health Questionnaire; Rumination = RSS-SF, 10-item Rumination Response Scale- Short Form; Cognitive Reappraisal and Suppression = ERQ, 10-item Emotion Regulation Questionnaire; Insomnia = ISI, 7-item Insomnia Severity Index; Mindfulness and facets = FFMQ-SF, 24-item Five Facets Mindfulness Questionnaire- Short Form. †*p* <0.10; **p* < 0.05; ***p* < 0.01; ****p* ≤0.001.


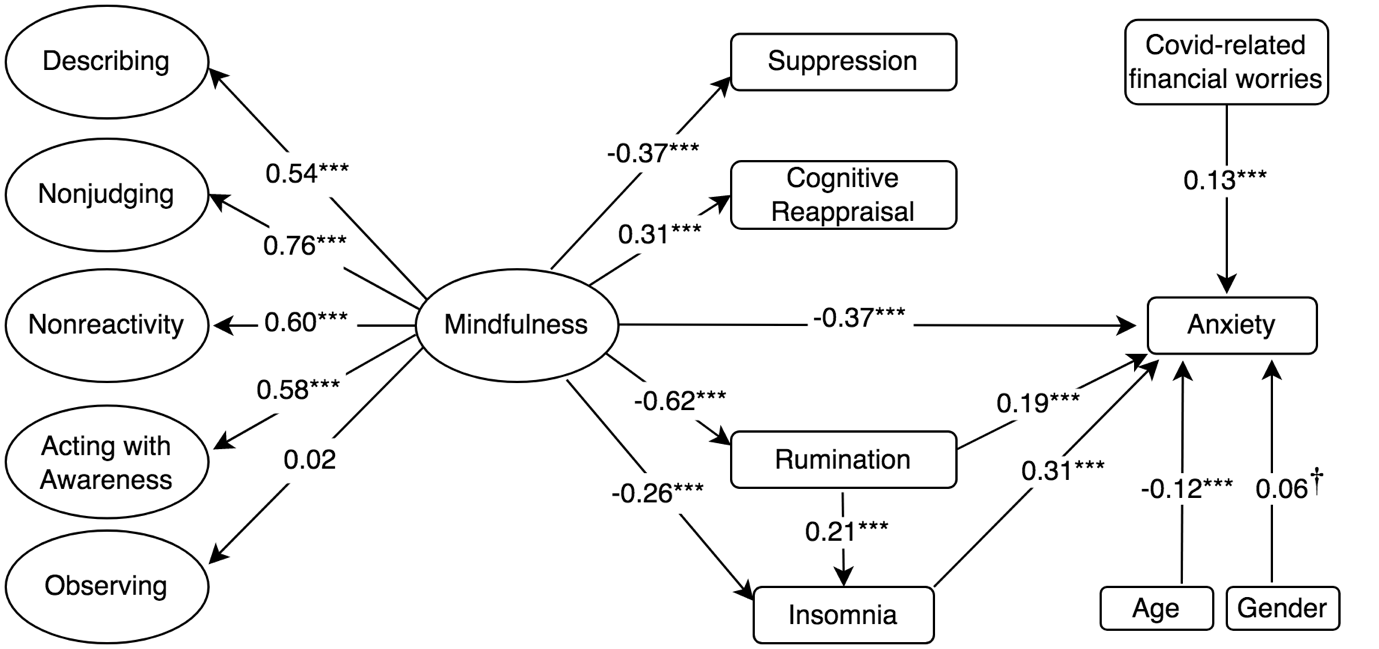


**Fig 8.** Standardized mediation Model 8 of the effect of higher-order mindfulness on anxiety. Anxiety = GAD-7, a 7-item Generalised Anxiety Disorder; Rumination = RSS-SF, 10-item Rumination Response Scale- Short Form; Cognitive Reappraisal and Suppression = ERQ, 10-item Emotion Regulation Questionnaire; Insomnia = ISI, 7-item Insomnia Severity Index; Mindfulness and facets = FFMQ-SF, 24-item Five Facets Mindfulness Questionnaire- Short Form. †*p* <0.10; **p* < 0.05; ***p* < 0.01; ****p* ≤0.001.


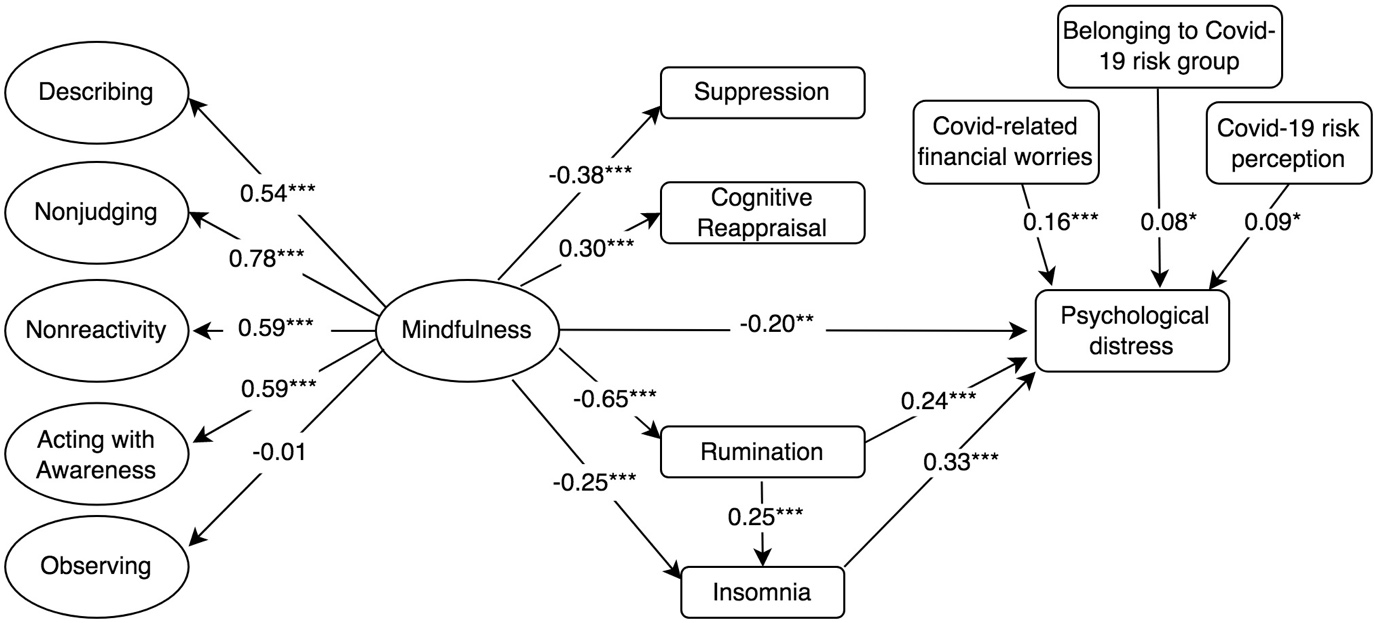


**Fig 9.** Standardized mediation Model 9 of the effect of higher-order mindfulness on COVID-related psychological distress. Psychological impact of COVID-19 = IES-R, 22-item Impact of Event Scale-Revised; Rumination = RSS-SF, 10-item Rumination Response Scale- Short Form; Cognitive Reappraisal and Suppression = ERQ, 10-item Emotion Regulation Questionnaire; Insomnia = ISI, 7-item Insomnia Severity Index; Mindfulness and facets = FFMQ-SF, 24-item Five Facets Mindfulness Questionnaire- Short Form. †p <0.10; *p < 0.05; **p < 0.01; ***p ≤0.001.


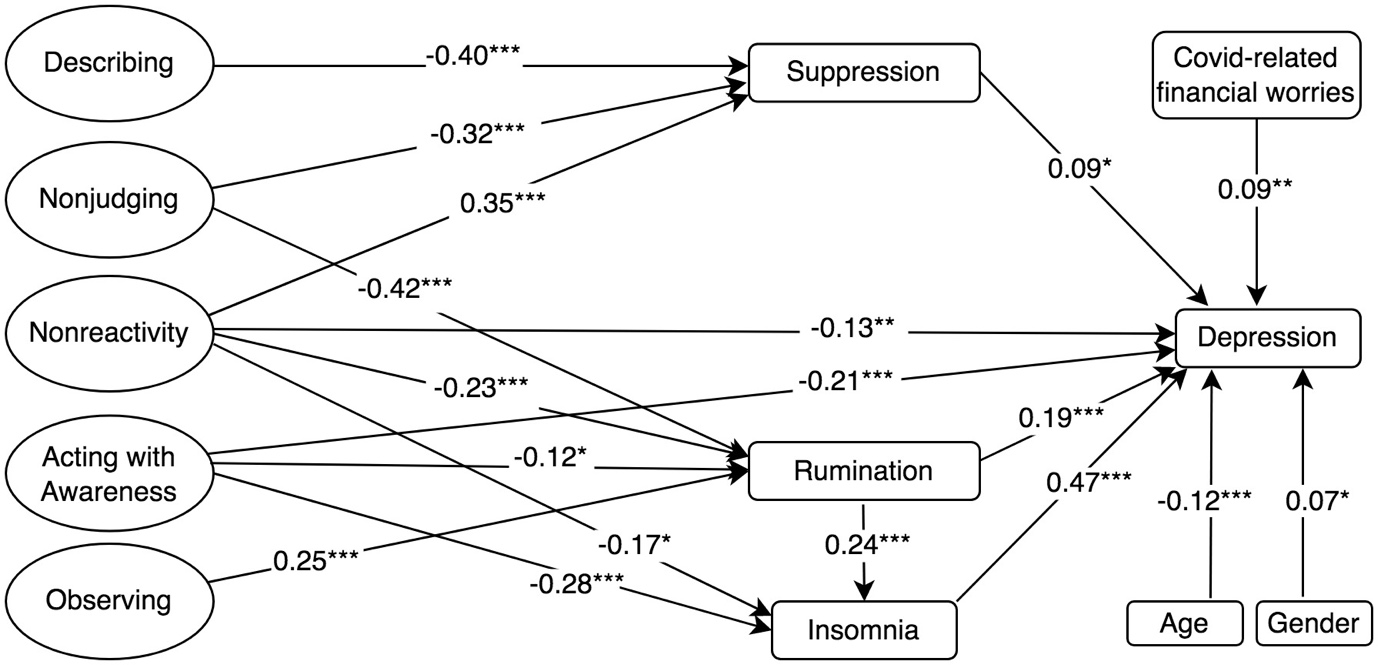


**Fig 10.** Standardized mediation Model 10 of the effect of the five-facets of mindfulness on depression. Depression = PHQ-8, 8-item Patient Health Questionnaire; Rumination = RSS-SF, 10-item Rumination Response Scale- Short Form; Cognitive Reappraisal and Suppression = ERQ, 10-item Emotion Regulation Questionnaire; Insomnia = ISI, 7-item Insomnia Severity Index; Mindfulness and facets = FFMQ-SF, 24-item Five Facets Mindfulness Questionnaire- Short Form. †*p* <0.10; **p* < 0.05; ***p* < 0.01; ****p* ≤0.001.


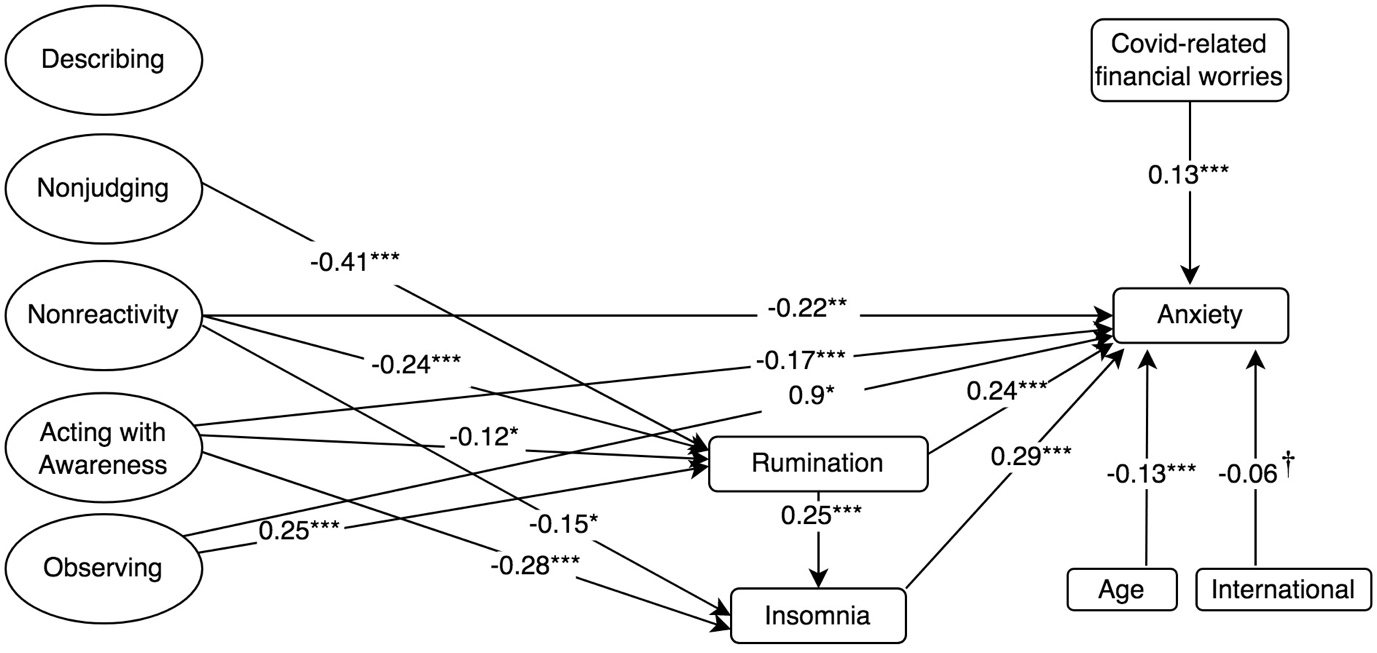


**Fig 11.** Standardized mediation Model 11 of the effect of the five-facets of mindfulness on anxiety. Anxiety = GAD-7, a 7-item Generalised Anxiety Disorder; Rumination = RSS-SF, 10-item Rumination Response Scale- Short Form; Cognitive Reappraisal and Suppression = ERQ, 10-item Emotion Regulation Questionnaire; Insomnia = ISI, 7-item Insomnia Severity Index; Mindfulness and facets = FFMQ-SF, 24-item Five Facets Mindfulness Questionnaire- Short Form. †*p* <0.10; **p* < 0.05; ***p* < 0.01; ****p* ≤0.001.


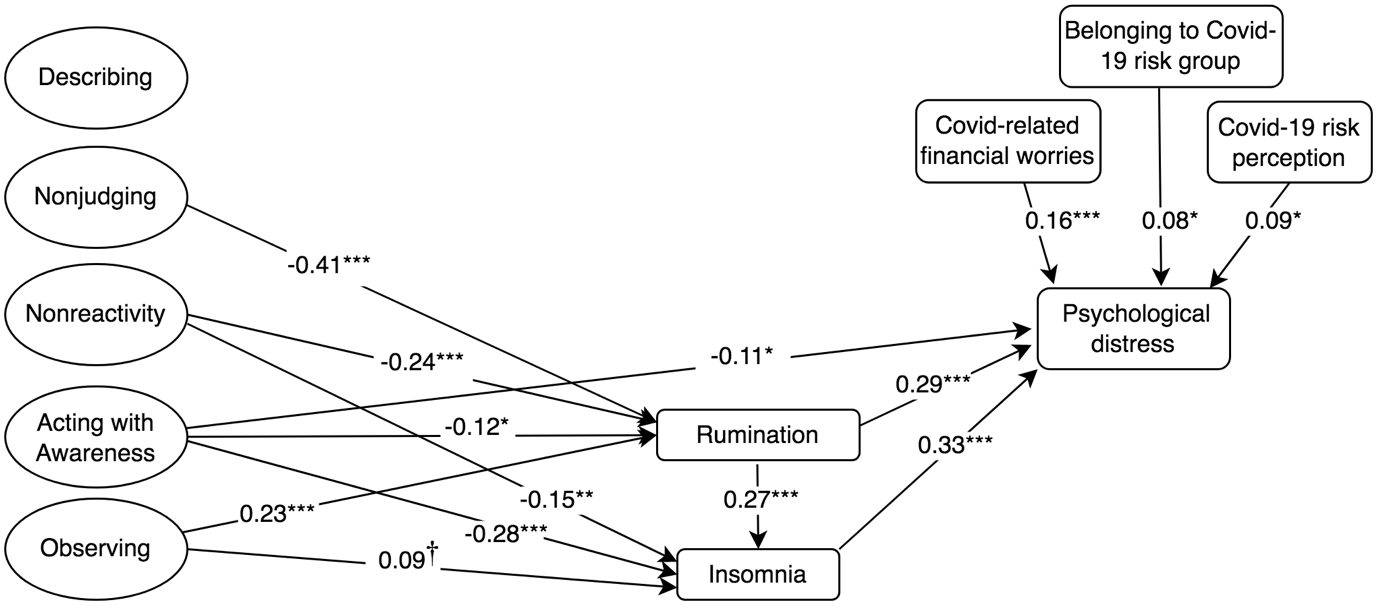


**Fig 12.** Standardized mediation Model 12 of the effect of the five-facets of mindfulness on COVID-related psychological distress. Psychological impact of COVID-19 = IES-R, 22-item Impact of Event Scale-Revised; Rumination = RSS-SF, 10-item Rumination Response Scale- Short Form; Cognitive Reappraisal and Suppression = ERQ, 10-item Emotion Regulation Questionnaire; Insomnia = ISI, 7-item Insomnia Severity Index; Mindfulness and facets = FFMQ-SF, 24-item Five Facets Mindfulness Questionnaire- Short Form. †p <0.10; *p < 0.05; **p < 0.01; ***p ≤0.001.
